# Not Just a Pain: A Medical Simulation Case About Biased Communication and Osteomyelitis in Pediatric Sickle Cell Anemia

**DOI:** 10.15766/mep_2374-8265.11335

**Published:** 2023-08-16

**Authors:** Adeola A. Kosoko, Yakira R. Alford, Karl A. Upplegger, Gowri S. Stevens

**Affiliations:** 1 Assistant Professor, Department of Emergency Medicine, McGovern Medical School at the University of Texas Health Science Center at Houston; 2 Third-Year Resident, Department of Emergency Medicine, McGovern Medical School at the University of Texas Health Science Center at Houston; 3 Pediatric Emergency Medicine Fellow, Department of Emergency Medicine, McGovern Medical School at the University of Texas Health Science Center at Houston

**Keywords:** Bias, Clinical Reasoning/Diagnostic Reasoning, Clinical Teaching/Bedside Teaching, Communication Skills, Emergency Medicine, Hematology, Simulation, Diversity, Equity, Inclusion

## Abstract

**Introduction:**

Biases in communication can be harmful to patient perceptions of care and the medical team's decision-making. Optimal communication must be taught and practiced similarly to the optimal management of the complex medical conditions associated with sickle cell disease (SCD). This simulation is designed to teach about biases, optimizing communication to and about a patient with SCD, and appropriately diagnosing and managing pediatric osteomyelitis as a complication of SCD.

**Methods:**

We designed and implemented a simulation case targeting emergency medicine residents and fellows to raise awareness about biases associated with SCD care and the complication of osteomyelitis in children with SCD. The case was delivered as a scheduled educational activity. Guided debriefing about optimizing care and communication for this patient population followed the simulation. We measured outcomes based on facilitator field notes and participant evaluations (Likert-scale and open-response questions).

**Results:**

Forty learners of varying medical practice proficiencies, societal experiences, and demographics participated, with 30 completing the postsimulation feedback survey. A majority (97%) of participants indicated that the experience was useful and would improve their clinical performance. Participants learned from each other's language and communication styles and reflected on their own communication.

**Discussion:**

Overall, participants found the simulation very useful as a review of the medical diagnosis and management of osteomyelitis in pediatric SCD. Moreover, they were very engaged and interested in the opportunity to learn about communication biases, particularly as these relate to SCD, to optimize their patient care.

## Educational Objectives

By the end of this activity, learners will be able to:

1. Medically optimize care for a patient with sickle cell anemia and resultant osteomyelitis.
2. Appropriately treat pain crisis in a patient with sickle cell anemia.
3. Explain the impact of providers’ language biases on patient care.
4. Develop strategies for discussing sickle cell anemia as a commonly stigmatized diagnosis in the clinical setting.

## Introduction

Bias (Table 1) can serve as a barrier to optimal health care within an emergency department (ED). Unfortunately, many biases are based on nonmodifiable factors such as age, race, or gender. Bias can be explicit or implicit. Explicit biases (Table 1) are conscious attitudes toward and preconceptions about a specific group. Explicit biases are often even taught to aid in quick and efficient decision-making. The utilization of explicit bias in the provision of health care may be particularly detrimental to marginalized patient populations, whose groups are associated with negative attitudes. Van Ryn and Burke demonstrated that physicians’ perceptions of a patient's intelligence, likelihood of engaging in high-risk behaviors, and medication noncompliance are associated with the patient's race and socioeconomic status (SES).^1^ Specifically, they found that physicians tend to perceive African Americans and members of low-SES groups generally more negatively than their White counterparts and members of high-SES groups.^1^

**Table 1.**
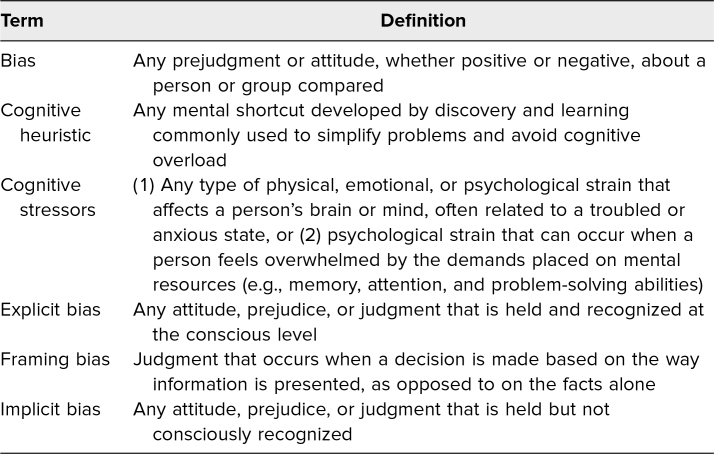
Glossary of Terms

Implicit bias (Table 1) refers to the subconscious attitude held by an individual toward a group of people. Health care providers in the ED often make decisions based on cognitive heuristics (Table 1) that are impacted by implicit bias due to limited patient information, time constraints, and frequent workflow interruptions. As such, cognitive stressors (Table 1) may increase implicit biases, particularly racial bias, which can negatively affect patient care.^2^ The effect of both implicit and explicit racial bias on patient care has led to health care disparities that are associated with poor outcomes among Black patients—a group already societally marginalized and medically disenfranchised. For instance, bias has been linked to a disproportionate increase in maternal morbidity rates and preterm births among Black patients.^3,4^ Specific to the ED, several studies have shown that Black patients are less likely to receive adequate analgesia than White patients.^5,6^

Such biases pose a pain management challenge for patients with sickle cell disease (SCD)—a disease that disproportionately affects patients of African descent (i.e., Black patients). The National Heart, Lung, and Blood Institute (NHLBI) has published guidelines regarding the management of the acute vaso-occlusive crisis, yet these guidelines have not been widely adopted.^7^ One study showed that 98% of ED physicians felt confident in how they managed sickle cell crises, yet only 24% were aware of the NHLBI guidelines.^8^ Other studies have shown that many patients presenting with vaso-occlusive crises believe their pain is poorly controlled or inadequately managed.^9,10^

Emergency medicine (EM) as a specialty continues to struggle to set standards for pain management in general due to the rise of misuse of the ED by people with analgesic addictions. As a result, patients with sickle cell anemia, who often present with some level of pain, often fall prey to suboptimal care based on faulty heuristics and preconceived notions regarding the intent of their ED visits. Although iatrogenic addiction to opioids is rare,^11^ a survey conducted by Shapiro, Benjamin, Payne, and Heidrich revealed that 53% of EM physicians and 23% of hematologists believed that one in five patients with SCD was addicted to analgesics.^12^

Patients with SCD, adults and children alike, have considered racial bias and health-related stigmas to be factors in the suboptimal management of sickle cell pain crises.^13–15^ Haywood and colleagues revealed that African Americans with SCD were more likely to feel as though their health care providers were not listening to them, did not show them respect, or did not spend enough time with them.^16^ Patients with SCD have also been found to feel as though their pain was discredited by health care providers. As a result, patients experiencing discrimination may resort to maladaptive coping mechanisms or delay seeking treatment for pain due to health-related stigma.^17–19^

Although pain in patients with SCD is often attributed to vaso-occlusive crises, it may be the presenting symptom of a life-threatening complication of SCD, such as acute chest syndrome, splenic sequestration, septic joint, or osteomyelitis. Studies have revealed challenges in distinguishing osteomyelitis from vaso-occlusive crises in pediatric patients due to their similar clinical presentations.^20,21^ Although patients with SCD are more likely to experience vaso-occlusive crises than osteomyelitis, pediatric patients with SCD have a significantly higher risk of developing osteomyelitis caused by salmonella than the general population.^22,23^ Therefore, it is imperative to consider osteomyelitis as a differential diagnosis and, in doing so, pursue appropriate diagnostic studies, address pain complaints, and appropriately manage the underlying condition.

The purpose of this simulation case is to explore and improve communication as a source of bias concerning SCD in the emergency room setting among both medical teams and patients with their family members. This case can encourage participants to have a healthy discussion with their colleagues about biased communication in the ED while observing others’ behavior and considering their own roles in upholding optimal standards of patient care. Additionally, this simulation can educate participants on identifying acute osteomyelitis and optimally managing it in pediatric patients as a complication of SCD.

## Methods

### Development

We developed this simulation to help develop learners’ systematic approaches to the evaluation of the unique needs of a pediatric patient with sickle cell anemia. Specifically, we wanted the simulation to help people develop strategies with which to assess and advocate for optimal communication practices when discussing sickle cell anemia, which is often stigmatized by health care providers in clinical settings. The targeted learners were EM residents and pediatric emergency medicine (PEM) fellows who were familiar with simulation as a part of their longitudinal training curriculum. As part of their curriculum, each month, the learners participated in two to three simulation cases for which they were not expected or requested to complete prework or prerequisite learning objectives. Therefore, each learner was already familiar with the major basic tenets of medical simulation (i.e., welcome and trust, orientation to the environment, the fiction contract, and confidentiality). The facilitators of the case were its authors, who on the day of the simulation presented with materials consisting of the case template (Appendices A and B), anticipated debrief topics with common clinical pitfalls and complementary materials (Appendix C), an educational outline of language biases within the clinical setting, an instructor evaluation form, and a case evaluation form.

Prior to commencing, learners were notified about the simulation case being evaluated for efficacy and about their participation being video recorded only for review by the authors/instructors. Learners were also notified that no personal identifying information would be utilized and that after the video had been reviewed for notes, it would be erased. Each participant volunteered, and there was no compensation. This project was deemed exempt by the University of Texas Health Science Center at Houston Institutional Review Board (HSC-MS-22-0635).

### Equipment

We administered the case in a high-fidelity simulation lab arranged to emulate a standard pediatric ED with a working space away from the patient and the patient room. We utilized a small, empty room to simulate a real clinical environment, similar to how residents and fellows might congregate in a workspace where they could receive clinical updates from nursing and medical students could give presentations.

Prior to the arrival of the learners, the necessary equipment was organized to mimic a clinical setting and consisted of a high-fidelity, child-like manikin and bedside equipment (pulse oximeter sensor and cardiac leads with vital signs monitor, oxygen access with nasal cannula adapter and rebreather mask, a bag-mask-valve, and intravenous line supplies). We utilized in-room technologies including PowerPoint and projection screens (television monitor, patient monitor) to provide laboratory reports, radiology results, and vital signs. If these technologies (televisions, a high-fidelity manikin, and patient monitors) are not available, a low-fidelity approach can be applied to the case (e.g., any manikin, printed or verbally reported vital signs, investigative study results, and clinical changes), copies of the results can be provided, and the facilitator can verbally communicate vital signs and clinical changes. A live, simulated patient can also be used in place of a manikin.

### Personnel

Our learner groups were meant to consist of four to six EM residents, pediatric residents, and/or PEM fellows (PGY 1-PGY 6). No more than one PEM fellow was in any group. There were no prerequisites for learners. Members of each group fulfilled the typical duties of a medical doctor in addition to minor bedside procedures and medication administration. The group was expected to participate as a team in providing optimal patient care.

An instructor/facilitator worked through the expected flow of the case, including managing the manikin, directing the learners, and providing or clarifying any information or troubleshooting.

#### At the physician workstation

A nurse standardized participant opened the case, briefly explaining the triage of the patient and providing the first iteration of biased language and opportunity for learners to address it. Thereafter, due to having other priorities in the department, the nurse was not available for bedside nursing, which the group was expected to manage on its own.

A medical student standardized participant then provided the original history obtained from the patient. The student's presentation provided the second iteration of biased language, as well as an opportunity to correct bias toward a patient with SCD and pain.

#### At the patient bedside

A parent standardized participant augmented the medical history (if asked) and also advocated for the care of their child. The parent respectfully asked to provide the history and answered questions for the child, who did not feel well enough to talk.

The case was written with four different standardized participants in mind, but we were able to adapt it to use three standardized participants differentiated by the following basic costumes:

• Nurse: name tag and medical scrubs.
• Medical student: waist-length white medical coat.
• Parent: sweater.
• General facilitator: primarily acting to voice any findings on exam, imaging, laboratory studies and as the voice of any requested consultants.

### Implementation

The simulation, including the time for debriefing, was 40 minutes long. The facilitator and the standardized participants were equipped with copies of the case details and debriefing prompts. The environment was prepared per the Equipment section above.

Prior to a group's interacting with the patient and the parent in the simulated patient room, the nurse and the medical student read their scripts to the group. The nurse and the medical student detailed the case, including triage from the nurse's point of view and the medical student's assessment of the patient, which were presumed to have occurred prior to the arrival of the group of learners (who acted as a team of preceptors to the medical student).

The learners played through the case with minimal interruptions, interacting among themselves and the necessary standardized participants. Learners began by diagnosing and treating the young child with sickle cell anemia who had an acute pain crisis due to osteomyelitis (Educational Objectives 1 and 2). The simulation ended at 20 minutes or earlier if the learners promptly declared a diagnosis, intervened, and implemented a disposition for the patient.

The participants assumed unfilled bedside roles and delegated tasks among themselves. One instructor (general facilitator) was responsible for displaying laboratory results, radiology images, and other diagnostics upon the team's request. This facilitator was also responsible for assisting in navigating portions of the simulation where the team reported confusion or misunderstanding as they maneuvered through different aspects of the case.

### Debriefing and Assessment

Facilitators ensured that at least 20 minutes remained for debriefing upon completion of thes case. Debriefing was facilitated using debriefing slides (Appendix C). The first portion of the debriefing process focused on pathophysiology of SCD, signs and symptoms of osteomyelitis, and preferred management and evaluation strategies of the child with osteomyelitis, SCD, and pain (Educational Objectives 1 and 2). The second portion of the debriefing process focused on communication with health care providers regarding patients with sickle cell anemia and their families (Educational Objectives 3 and 4).

For feedback, we used the plus-delta model, which encouraged the learners to summarize the case and acknowledge successful approaches and shortcomings in the applied strategies. We chose the plus-delta model because the primary focus of the debriefing was addressing communication areas of improvement without being overly judgmental about less-desirable communication when evaluating a system of care for a patient with SCD. The plus-delta model allowed for self-reflection on performance, and communication in this instance was more impactful for how an individual would respond in vivo. We also felt this method provided room for psychological safety for our participants. Plus-delta debriefing asked learners to reflect on the performance of the team, then to consider their performance as an individual within the team, by asking about what went well followed by what were areas for positive change.^24^ It was extremely important to us that our learners felt psychologically safe to make a mistake in the simulation and to look forward from it with a growth mindset rather than feeling a punitive weight. The facilitator provided direct feedback regarding the medical decision-making applied by the team versus current preferred medical approaches to pediatric SCD and osteomyelitis.^25^ Appendix C aided in teaching the learners about bias in communication. The facilitator did not directly provide individuals feedback on their communication. Rather, the facilitator prompted the group to reflect on personal communication and the communication of their colleagues, allowing the group to critique itself.

During the simulation and debriefing, one of the facilitators (e.g., the nurse standardized participant during our simulation) was tasked with taking field notes about observations of the behaviors of the groups and individuals. Each of the simulation groups was also videotaped (with verbal permission) for the remainder of the facilitators to review in order to similarly take field notes based on observations of behaviors. No personal identifiers were noted or reported. The video was stored on encrypted software. Immediately following the completion of the simulation, each learner participant in the medical simulation was sent a survey eliciting feedback about the simulation, as well as takeaways from the simulation and instructor evaluation.

## Results

**Figure 1.**
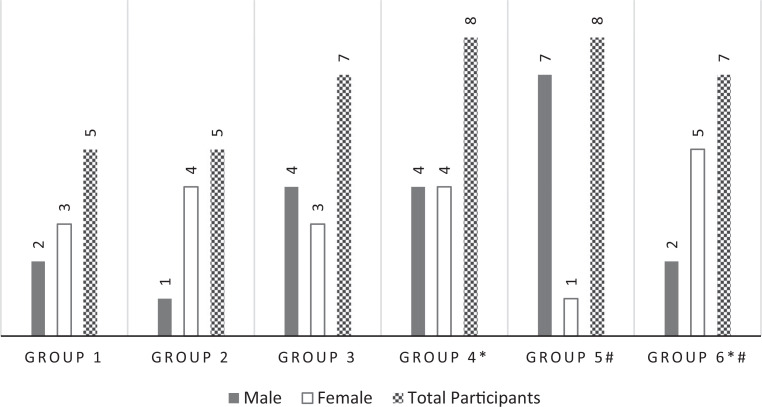
Simulation group distribution by gender. Asterisk (*) indicates pediatric emergency medicine fellow participation; pound sign (#) indicates medical student participation.

We implemented the simulation in a large 3-year residency program over several hours in a single day. We had a total of 40 participants ranging from fourth-year medical students to PEM fellows (PGY 1-PGY 6). Participants (females, *n* = 20) were divided into six groups, with five to eight learners randomly assigned to each group (Figure 1). The groups each represented diversity in training as well as in age, gender, sexual identification, race, ethnicity, and religion based on residency demographic data. Unfortunately, for each round of the simulation case, only 20 minutes were budgeted for debriefing. Although there was robust conversation, addressing all the intended points often felt rushed and more commonly required closer to 30 minutes. In addition, though we had designed the simulation for four to six learners per group, many residents arrived on location such that the later groups were slightly larger. Medical students were not anticipated in the original design, but they presented as part of their coursework, were interested, consented, and wanted to be involved, making the groups up to eight persons in size.

**Figure 2.**
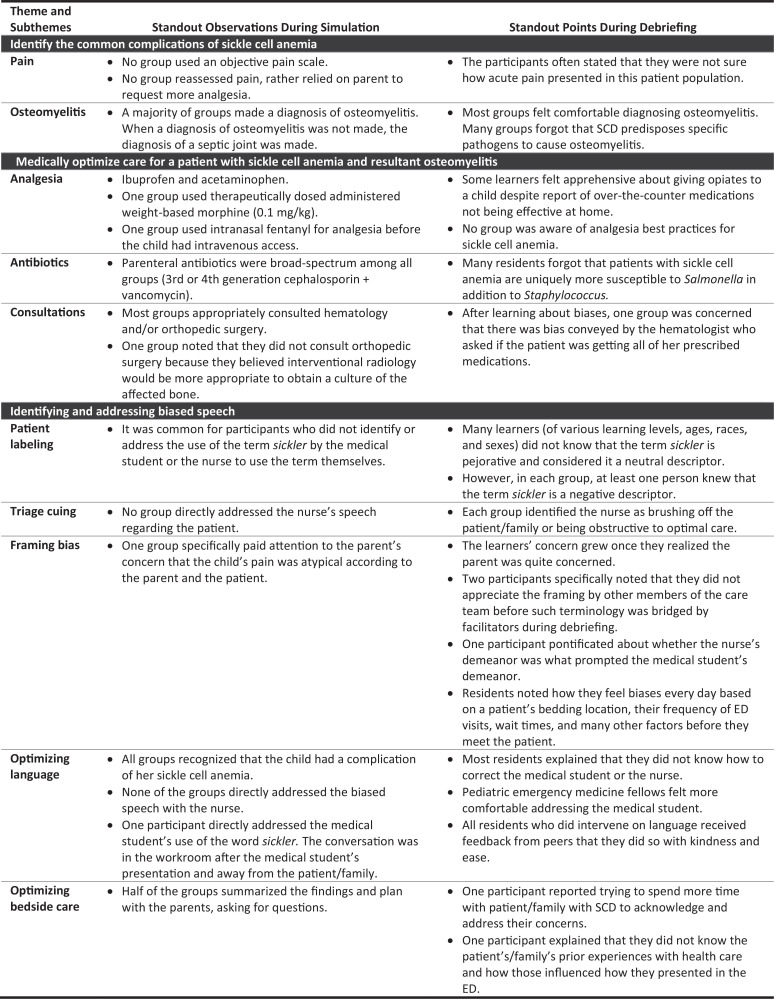
Standout researcher observations during simulations and debriefing sessions. Abbreviations: ED, emergency department; SCD, sickle cell disease.

Participants were asked to give feedback on the simulation case by using a quick response (QR) code provided at the conclusion of the debriefing session. The survey consisted of six questions. The first three questions elicited quantitative Likert-scale feedback on the medical simulation as a learning experience (Table 2). The next two questions were open-ended and solicited feedback about the simulation case, while the final question requested demographic categorization. Respondents were also asked to give feedback on how the experience could be improved and to describe the strengths of the case in open responses. Only EM residents opted in with responses using the QR code. Still, there was a paucity of open-ended responses in total (*n* = 9), which can be summarized as saying the simulation was a “great case” with a “good debrief” and “great leaders.” One learner specified appreciating that the simulation “touch[ed] on multiple topics [regarding] patient management,” and another learner stated, “It covered both a relevant clinical case as well as a review of bias.” One participant summarized the simulation feedback by saying, “We spend so much time studying medicine. It is also important to address factors that affect the way we practice medicine such as bias. I think this was one of the best medical simulations we've had.” Lastly, each researcher contributed field notes (general observations) about each of the iterations of the simulation case through live note-taking and reviewing videos to evaluate how well the course objectives were met (Figure 2).

**Table 2.**
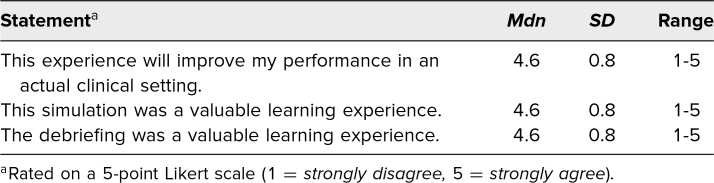
Quantitative Feedback (*N* = 30)

## Discussion

This medical simulation allowed us to take a novel approach to optimizing medical care for a child with SCD and resultant osteomyelitis by considering the physiologic factors in this patient group that require particular attention in addition to the societal factors that can complicate patient care. We believe, based on our experience with implementing this simulation, that it facilitates multiple discussions and learning opportunities specifically regarding pediatric sickle cell anemia, pain management, osteomyelitis, and bias and communication in medicine.

Language is a learned phenomenon. Many of our participants had learned the term *sickler* at some point in their medical careers, but only about half of them had learned that though a commonly used term, it can be received as a pejorative by the patients to whom it refers and, thereby, can cause distrust between the patient/family and the medical care team. Similarly, the casual dismissal of a patient's concern can occur in speech and can bias a physician or even multiple care team members against the consideration of serious medical pathologies. Based on the participant feedback and observations of discussions among learners, we gather that this simulation case is a valuable tool for teaching against biased communication among health care team members insofar as it challenges learners to consider how they will identify and address biased and derogatory communication in future in vivo scenarios.

Participants particularly appreciated that the lessons on bias and communication did not neglect that most of the patients with SCD present with medical complaints and it is the responsibility of the provider to appropriately diagnose and treat them medically. The integration of many of the nuances (e.g., triage, bias, communication, chronic pain, acute pain, complications, etc.) of treating a child with SCD complicated by osteomyelitis provided learning opportunities for trainees at varying stages in a safe environment.

Among our limitations was that we did not have each participant self-identify regarding individual demographics. We relied solely on demographics reported by candidates to the residency when they were applying via the Electronic Residency Application Service to determine that our participant sample was a diverse group based on many of the demographic markers. In this way, we did not make any evaluations about which groups were prone to have what knowledge about SCD, terminology, and biases. However, by not identifying individuals based on demographics, we also believed we were protecting personal identifiers. We were also colleagues of the learners, which may have contributed to biased responses from participants.

Anticipated group sizes for the simulation were five to six learners, whereas, because we had ongoing sessions, the groups became bigger with time, and our actual group sizes were five to eight learners. In general, medical simulation tends to be most successful with smaller group sizes because it encourages more active learner participation. The observers did not specifically note that there was less participation by learners in larger groups; however, group size could have limited some of the feedback we received or limited individual participation during debriefing sessions. Similarly, we had not anticipated the participation of medical students as learners when designing the simulation. The simulation, after all, does use a confederate in the role of a medical student. Medical student involvement was limited to the larger groups and was likely relegated to more of an observational role. In this type of simulation, due to intrinsic medical hierarchies, it might be difficult for a medical student to speak freely. Nonetheless, during debriefing, one student specifically noted that the exercise allowed them to observe how a student's presentation can impact a patient's care.

We relied on survey responses to determine learners’ perceptions of the simulation case. Participation was not mandatory, and we only captured about 70% of participants’ responses. We learned about participant perceptions of the simulation but were not able to measure behavior change or knowledge retention.

Future iterations could adjust the case based on learner feedback or facilitator observations (e.g., budgeting more time for debriefing). It would even be helpful to evaluate the same group experiencing the same simulation at a later date to evaluate for behavior change or retention of knowledge. Future researchers could also evaluate participant propensity to identify and intervene when biased language is used in future medical simulations or in vivo through chart review, searching for key terms. Other simulations could be developed to explore other areas in which there is bias or misunderstanding about the social implications of patient care and how a provider can best navigate medical complications and social factors.

Learners who participated in this medical simulation were afforded a unique opportunity to explore the diagnosis and management of osteomyelitis in a child with SCD while considering how societal implementations in the form of biased communication can affect patient care. Participant feedback suggests that the information provided by this simulation case is applicable and true to the provider and patient/family experience and relationship.

## Appendices


Simulation Case.docxSimulation Stimuli.pptxDebriefing Materials.pptx
*All appendices are peer reviewed as integral parts of the Original Publication.*
