# Supplementary material for: Not Just a Pain: A Medical Simulation Case About Biased Communication and Osteomyelitis in Pediatric Sickle Cell Anemia
Source: MedEdPORTAL. 2023 Aug 16;19:11335. doi: 10.15766/mep_2374-8265.11335 (PMC10427742; doi:10.15766/mep_2374-8265.11335)
Supplement: Supplementary file 1 — Simulation Case.docxSimulation Stimuli.pptxDebriefing Materials.pptx [file mep_2374-8265.11335-s001.zip › B. Simulation Stimuli.pptx]

## Slide 1
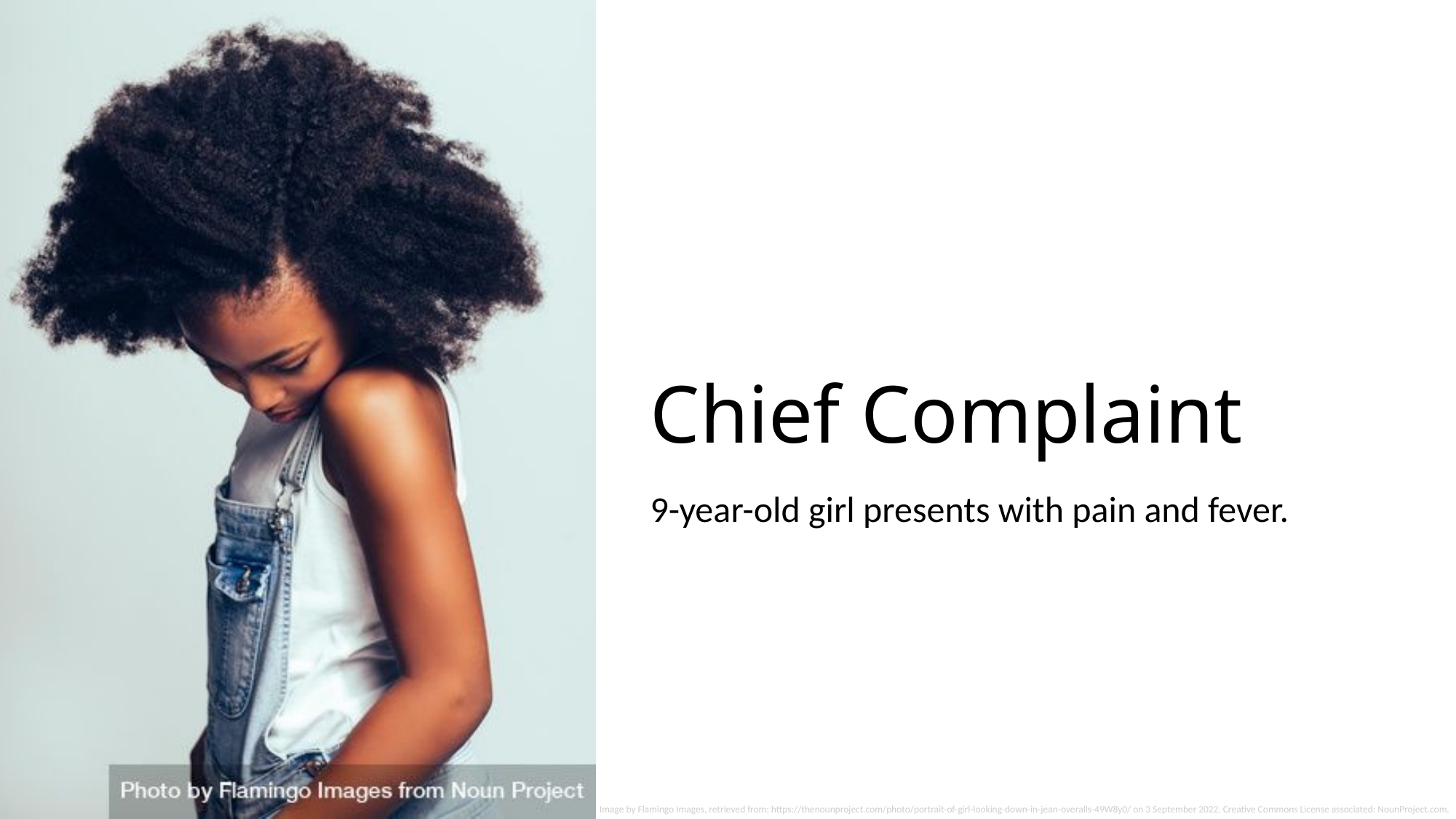

# Chief Complaint
9-year-old girl presents with pain and fever.
Image by Flamingo Images, retrieved from: https://thenounproject.com/photo/portrait-of-girl-looking-down-in-jean-overalls-49W8y0/ on 3 September 2022. Creative Commons License associated: NounProject.com.

## Slide 2
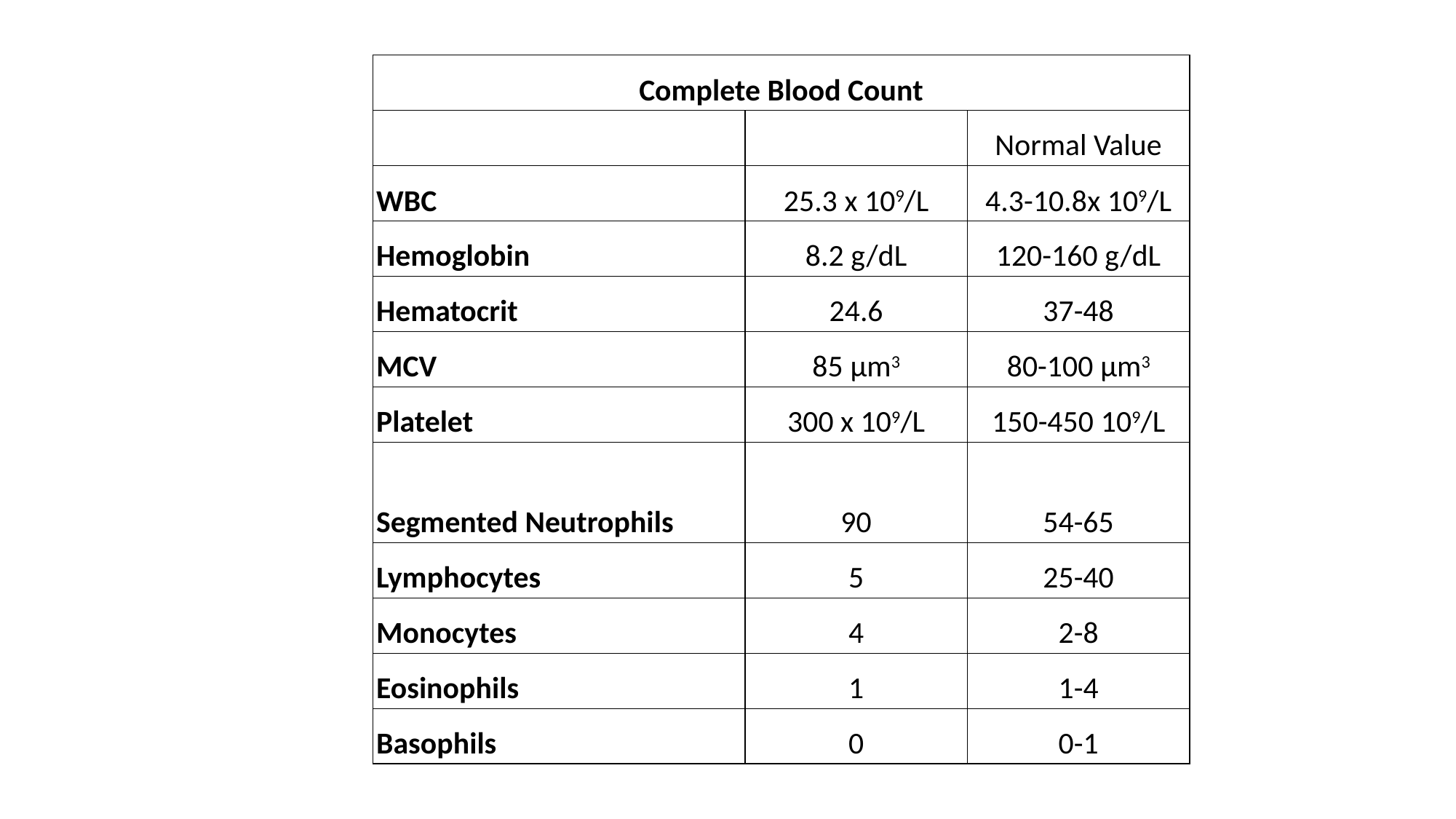

| Complete Blood Count | | |
| --- | --- | --- |
| | | Normal Value |
| WBC | 25.3 x 109/L | 4.3-10.8x 109/L |
| Hemoglobin | 8.2 g/dL | 120-160 g/dL |
| Hematocrit | 24.6 | 37-48 |
| MCV | 85 µm3 | 80-100 µm3 |
| Platelet | 300 x 109/L | 150-450 109/L |
| Segmented Neutrophils | 90 | 54-65 |
| Lymphocytes | 5 | 25-40 |
| Monocytes | 4 | 2-8 |
| Eosinophils | 1 | 1-4 |
| Basophils | 0 | 0-1 |

## Slide 3
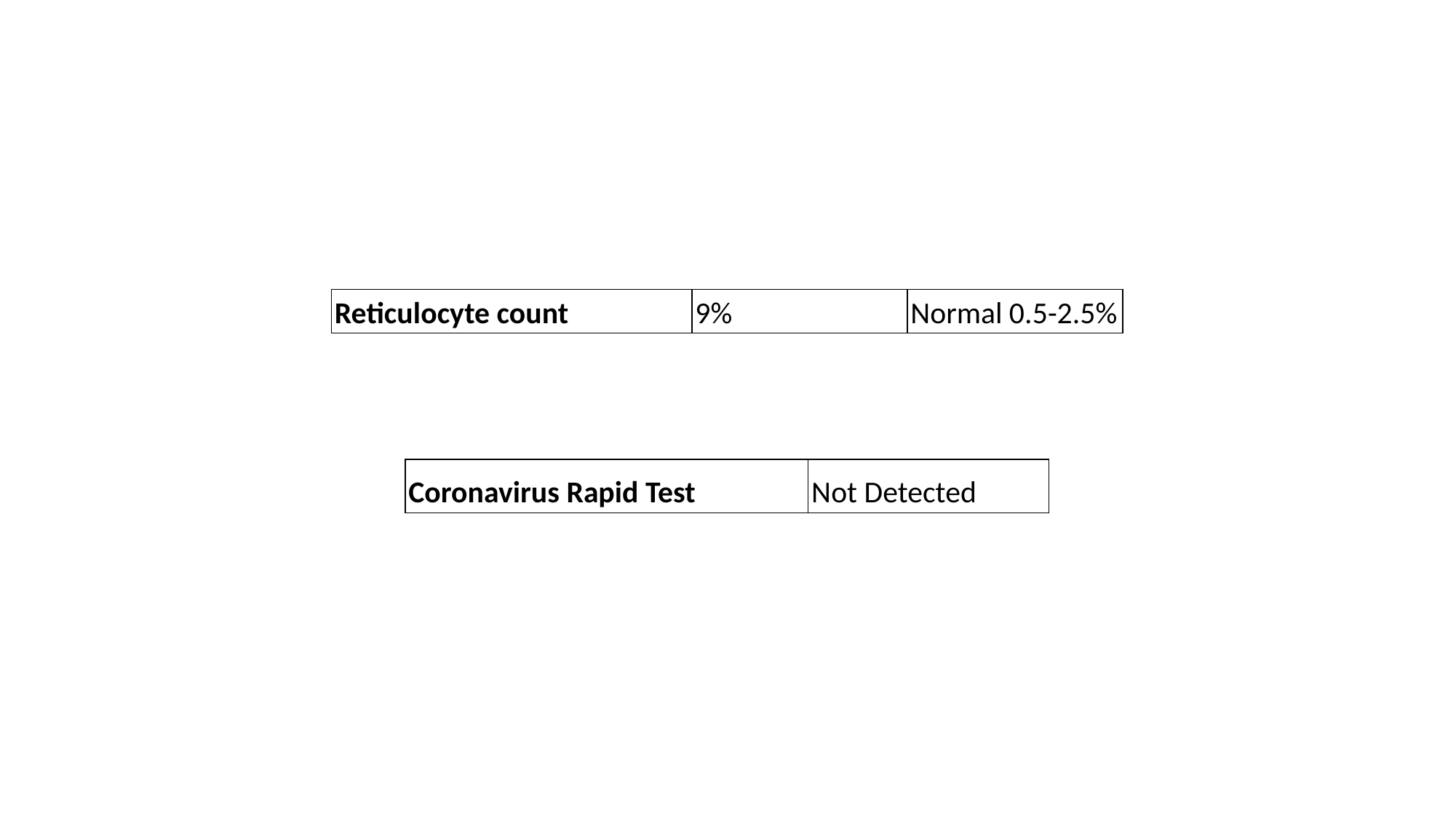

| Reticulocyte count | 9% | Normal 0.5-2.5% |
| --- | --- | --- |
| Coronavirus Rapid Test | Not Detected |
| --- | --- |

## Slide 4
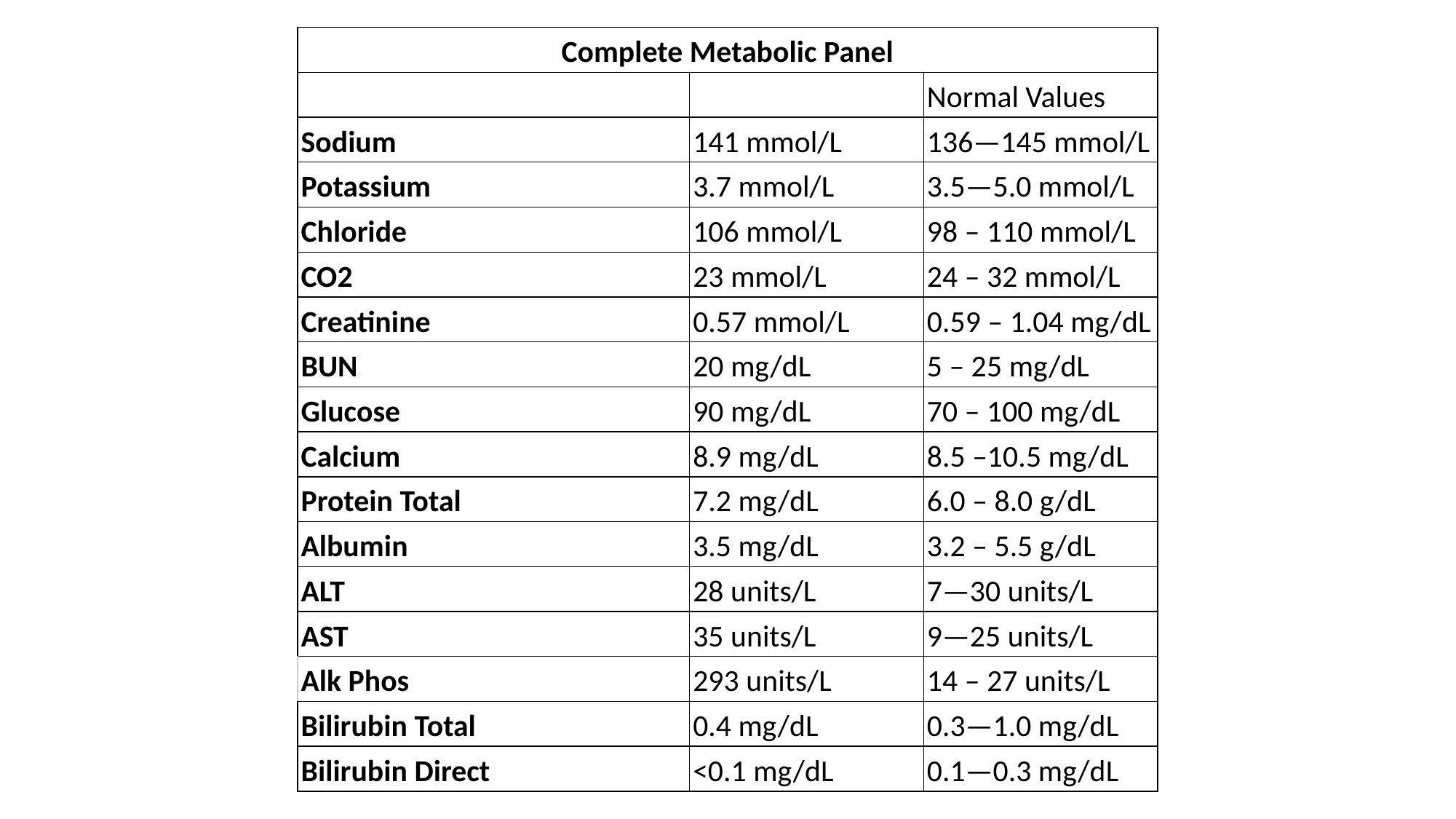

| Complete Metabolic Panel | | |
| --- | --- | --- |
| | | Normal Values |
| Sodium | 141 mmol/L | 136—145 mmol/L |
| Potassium | 3.7 mmol/L | 3.5—5.0 mmol/L |
| Chloride | 106 mmol/L | 98 – 110 mmol/L |
| CO2 | 23 mmol/L | 24 – 32 mmol/L |
| Creatinine | 0.57 mmol/L | 0.59 – 1.04 mg/dL |
| BUN | 20 mg/dL | 5 – 25 mg/dL |
| Glucose | 90 mg/dL | 70 – 100 mg/dL |
| Calcium | 8.9 mg/dL | 8.5 –10.5 mg/dL |
| Protein Total | 7.2 mg/dL | 6.0 – 8.0 g/dL |
| Albumin | 3.5 mg/dL | 3.2 – 5.5 g/dL |
| ALT | 28 units/L | 7—30 units/L |
| AST | 35 units/L | 9—25 units/L |
| Alk Phos | 293 units/L | 14 – 27 units/L |
| Bilirubin Total | 0.4 mg/dL | 0.3—1.0 mg/dL |
| Bilirubin Direct | <0.1 mg/dL | 0.1—0.3 mg/dL |

## Slide 5
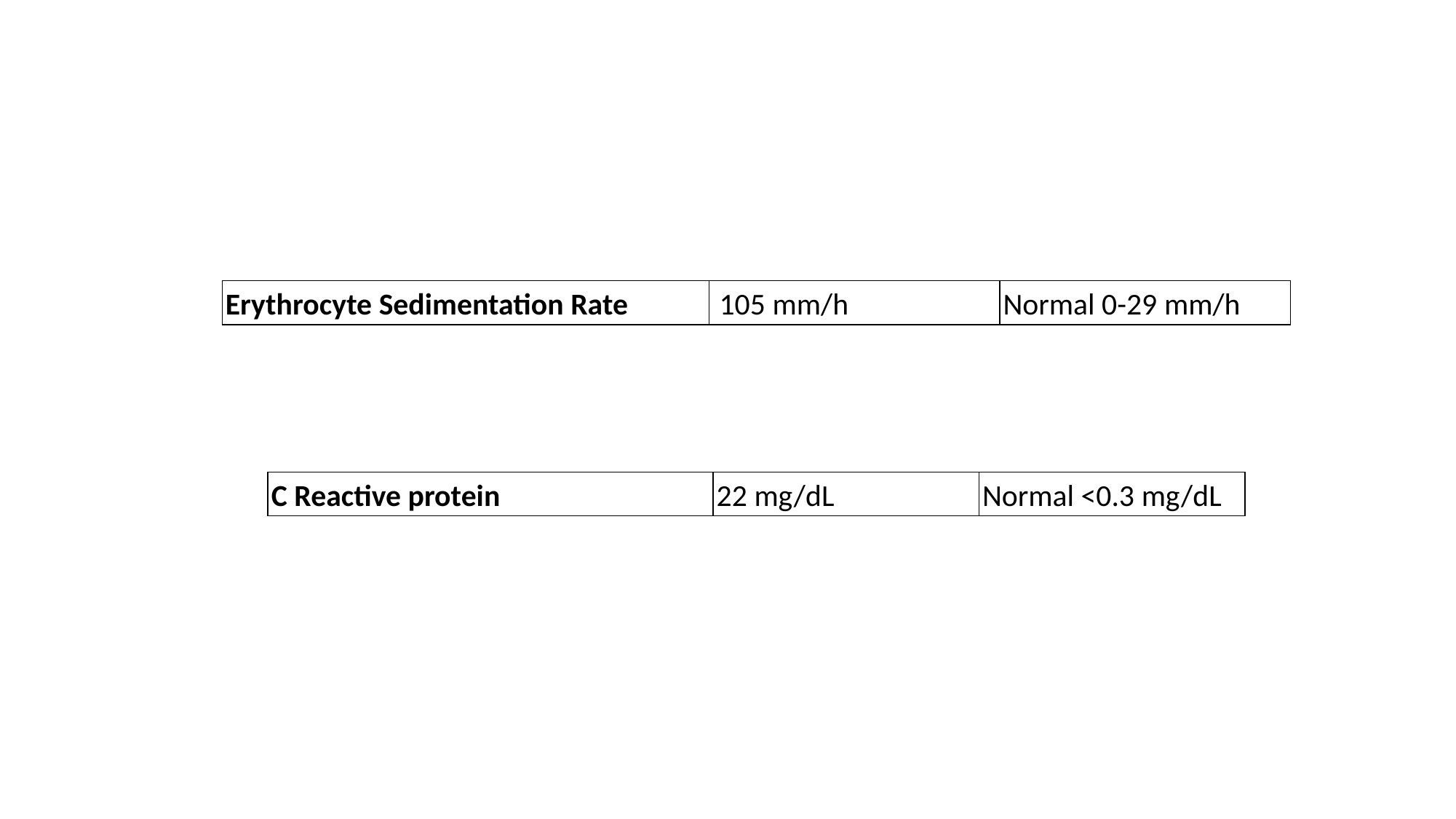

| Erythrocyte Sedimentation Rate | 105 mm/h | Normal 0-29 mm/h |
| --- | --- | --- |
| C Reactive protein | 22 mg/dL | Normal <0.3 mg/dL |
| --- | --- | --- |

## Slide 6
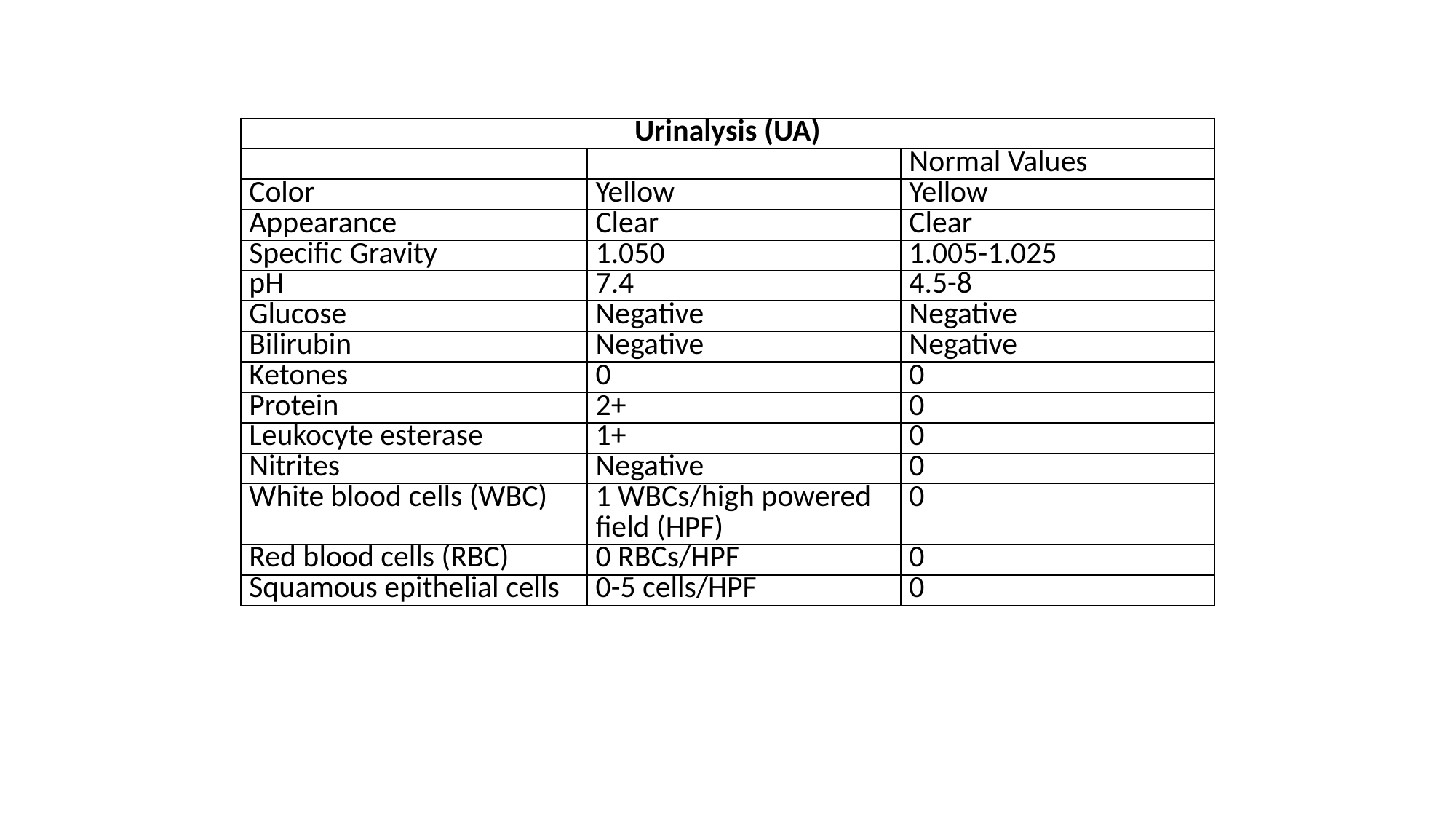

| Urinalysis (UA) | | |
| --- | --- | --- |
| | | Normal Values |
| Color | Yellow | Yellow |
| Appearance | Clear | Clear |
| Specific Gravity | 1.050 | 1.005-1.025 |
| pH | 7.4 | 4.5-8 |
| Glucose | Negative | Negative |
| Bilirubin | Negative | Negative |
| Ketones | 0 | 0 |
| Protein | 2+ | 0 |
| Leukocyte esterase | 1+ | 0 |
| Nitrites | Negative | 0 |
| White blood cells (WBC) | 1 WBCs/high powered field (HPF) | 0 |
| Red blood cells (RBC) | 0 RBCs/HPF | 0 |
| Squamous epithelial cells | 0-5 cells/HPF | 0 |

## Slide 7
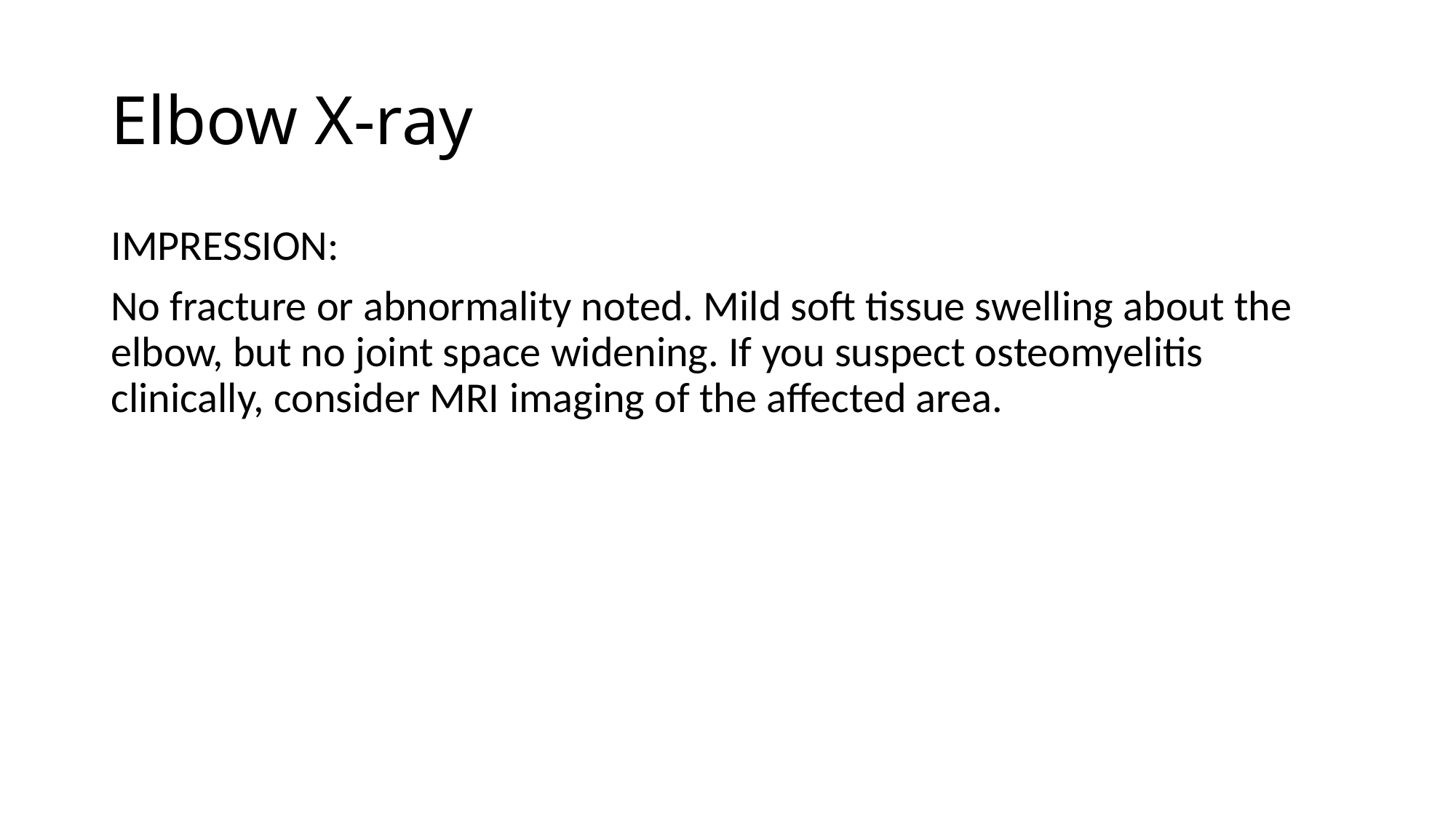

# Elbow X-ray
IMPRESSION:
No fracture or abnormality noted. Mild soft tissue swelling about the elbow, but no joint space widening. If you suspect osteomyelitis clinically, consider MRI imaging of the affected area.
