# Supplementary material for: Not Just a Pain: A Medical Simulation Case About Biased Communication and Osteomyelitis in Pediatric Sickle Cell Anemia
Source: MedEdPORTAL. 2023 Aug 16;19:11335. doi: 10.15766/mep_2374-8265.11335 (PMC10427742; doi:10.15766/mep_2374-8265.11335)
Supplement: Supplementary file 1 — Simulation Case.docxSimulation Stimuli.pptxDebriefing Materials.pptx [file mep_2374-8265.11335-s001.zip › C. Debriefing Materials.pptx]

## Slide 1
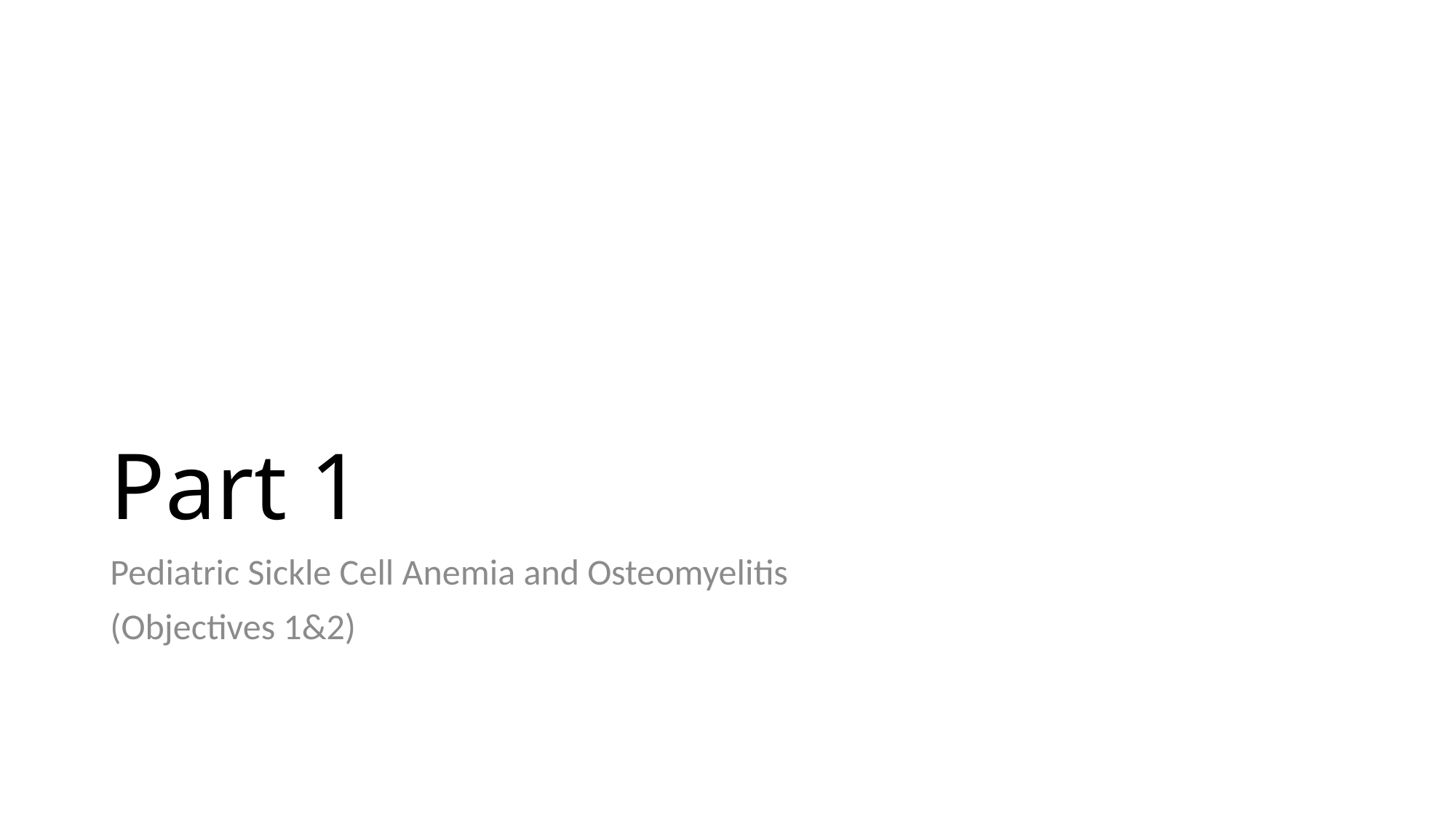

# Part 1
Pediatric Sickle Cell Anemia and Osteomyelitis
(Objectives 1&2)

## Slide 2
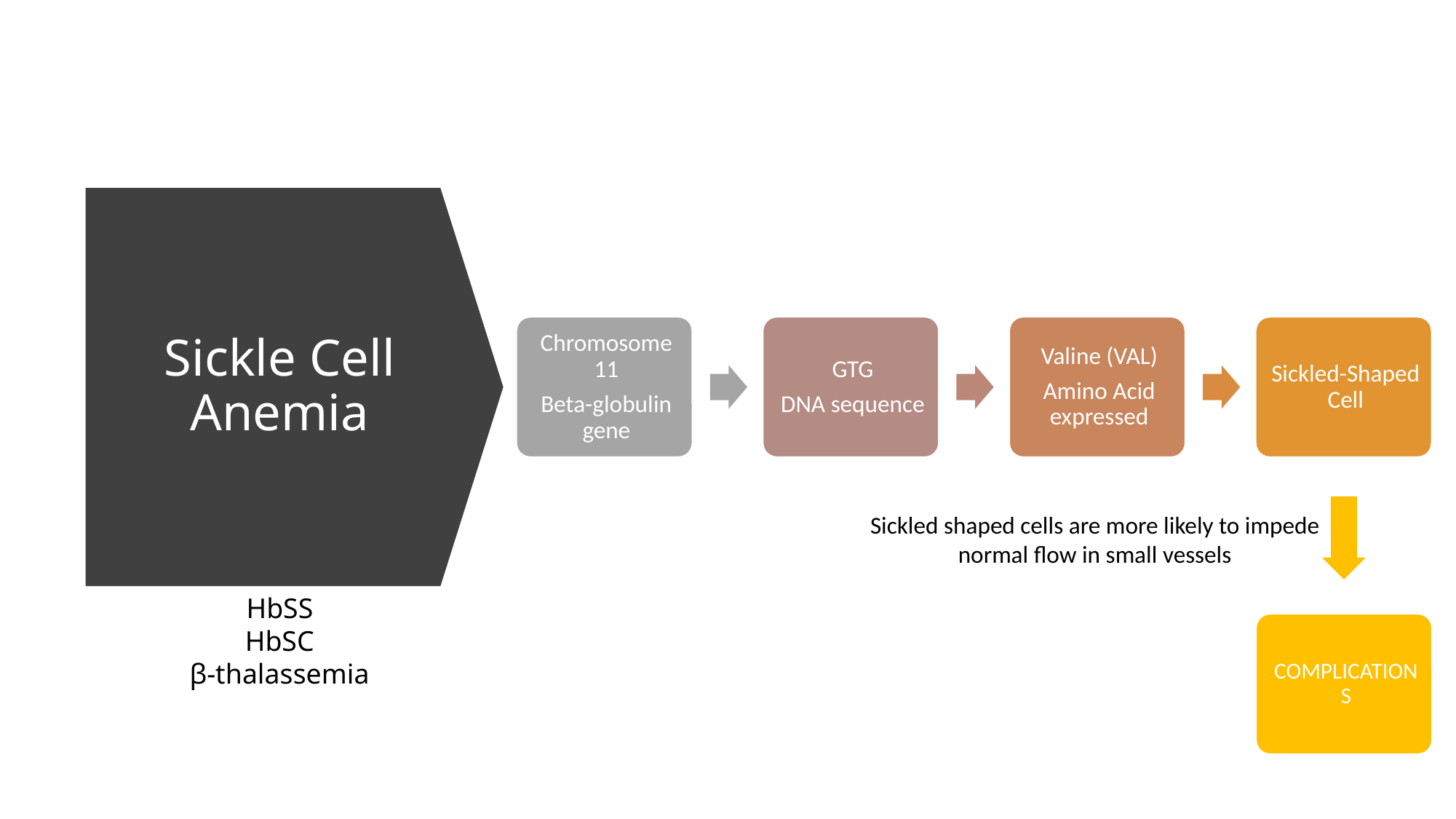

# Sickle Cell Anemia
Sickled shaped cells are more likely to impede normal flow in small vessels
HbSS
HbSC
β-thalassemia

## Slide 3
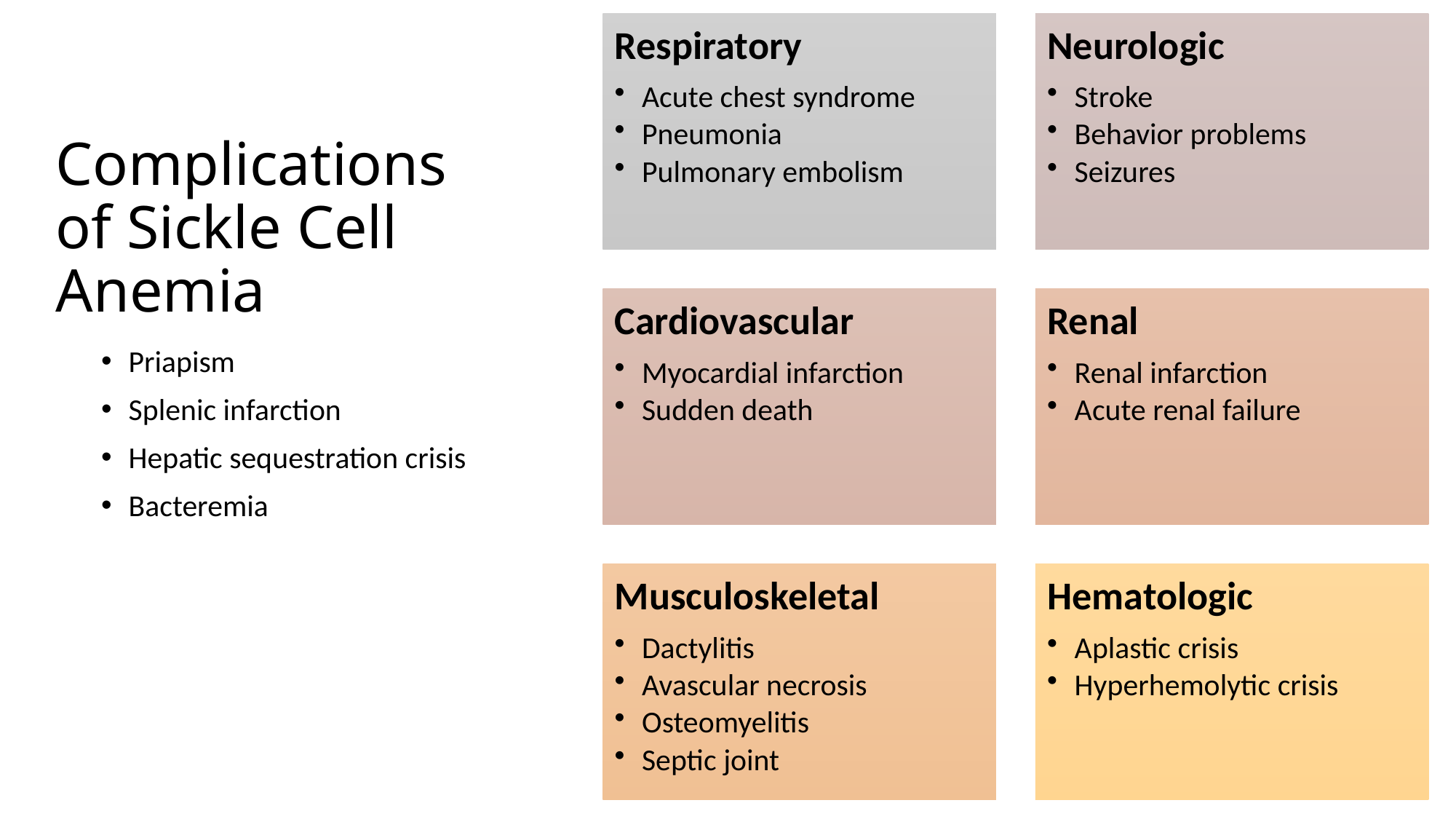

# Complications of Sickle Cell Anemia
Priapism
Splenic infarction
Hepatic sequestration crisis
Bacteremia

## Slide 4
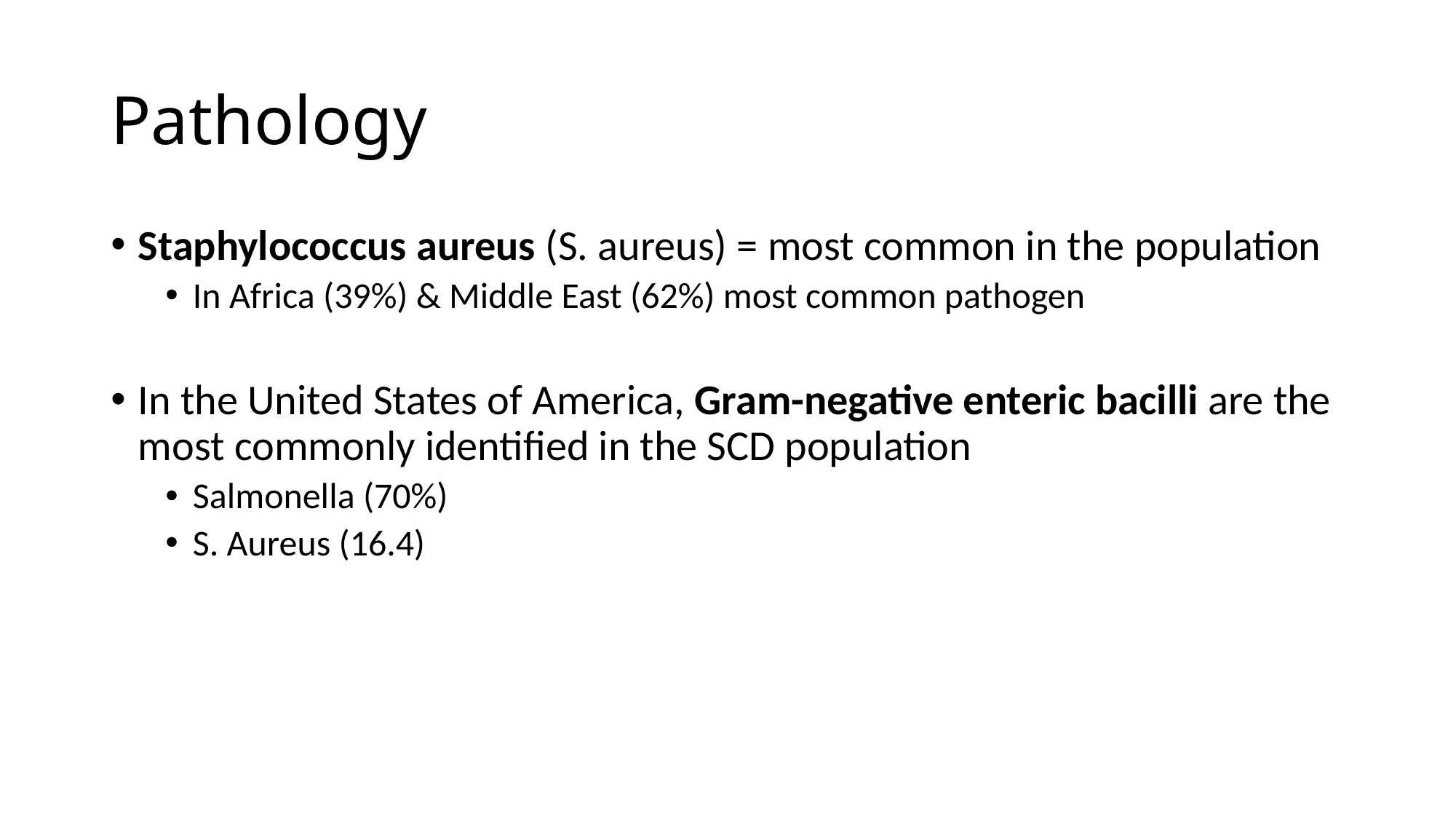

# Pathology
Staphylococcus aureus (S. aureus) = most common in the population
In Africa (39%) & Middle East (62%) most common pathogen
In the United States of America, Gram-negative enteric bacilli are the most commonly identified in the SCD population
Salmonella (70%)
S. Aureus (16.4)

## Slide 5
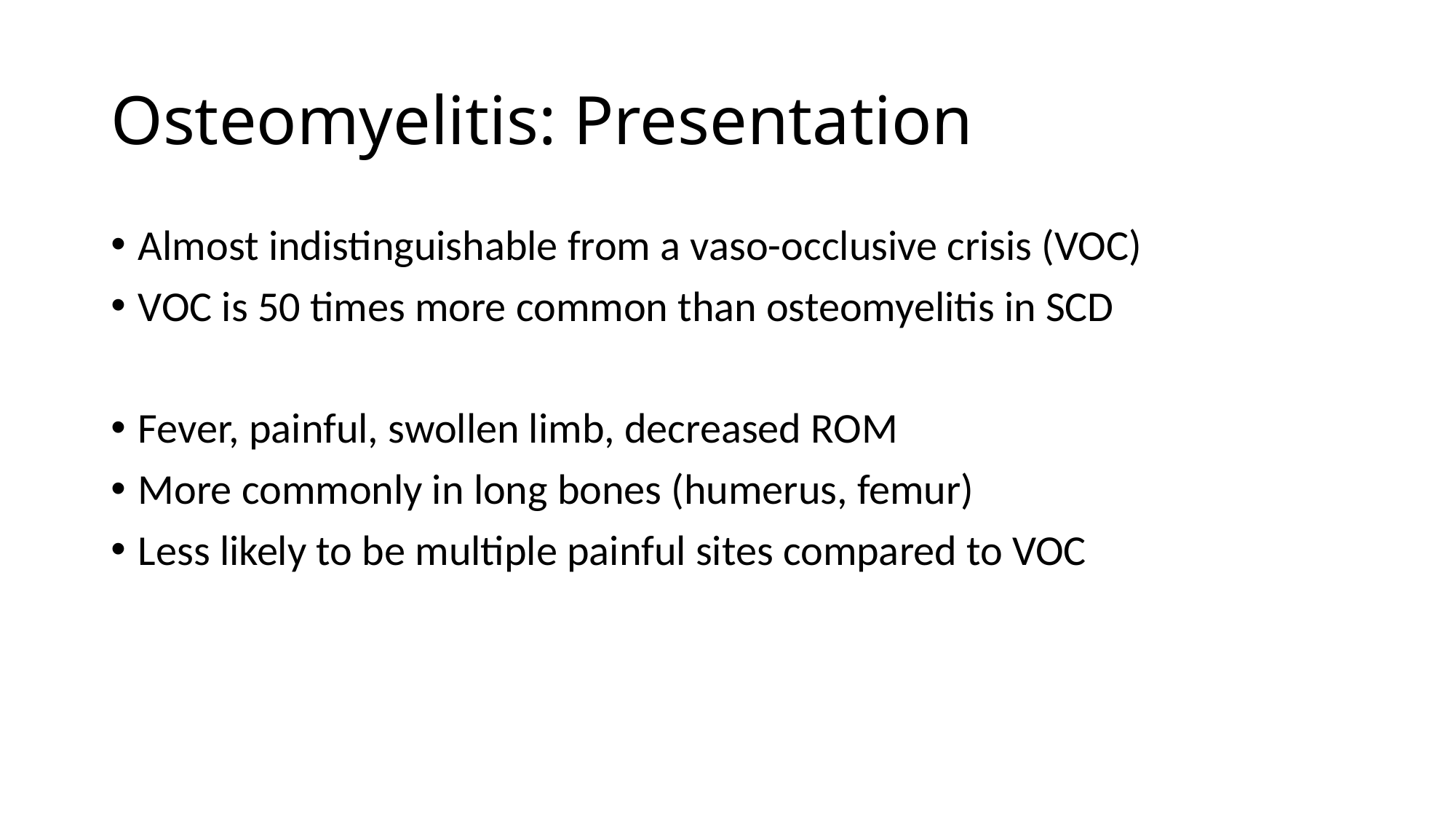

# Osteomyelitis: Presentation
Almost indistinguishable from a vaso-occlusive crisis (VOC)
VOC is 50 times more common than osteomyelitis in SCD
Fever, painful, swollen limb, decreased ROM
More commonly in long bones (humerus, femur)
Less likely to be multiple painful sites compared to VOC

## Slide 6
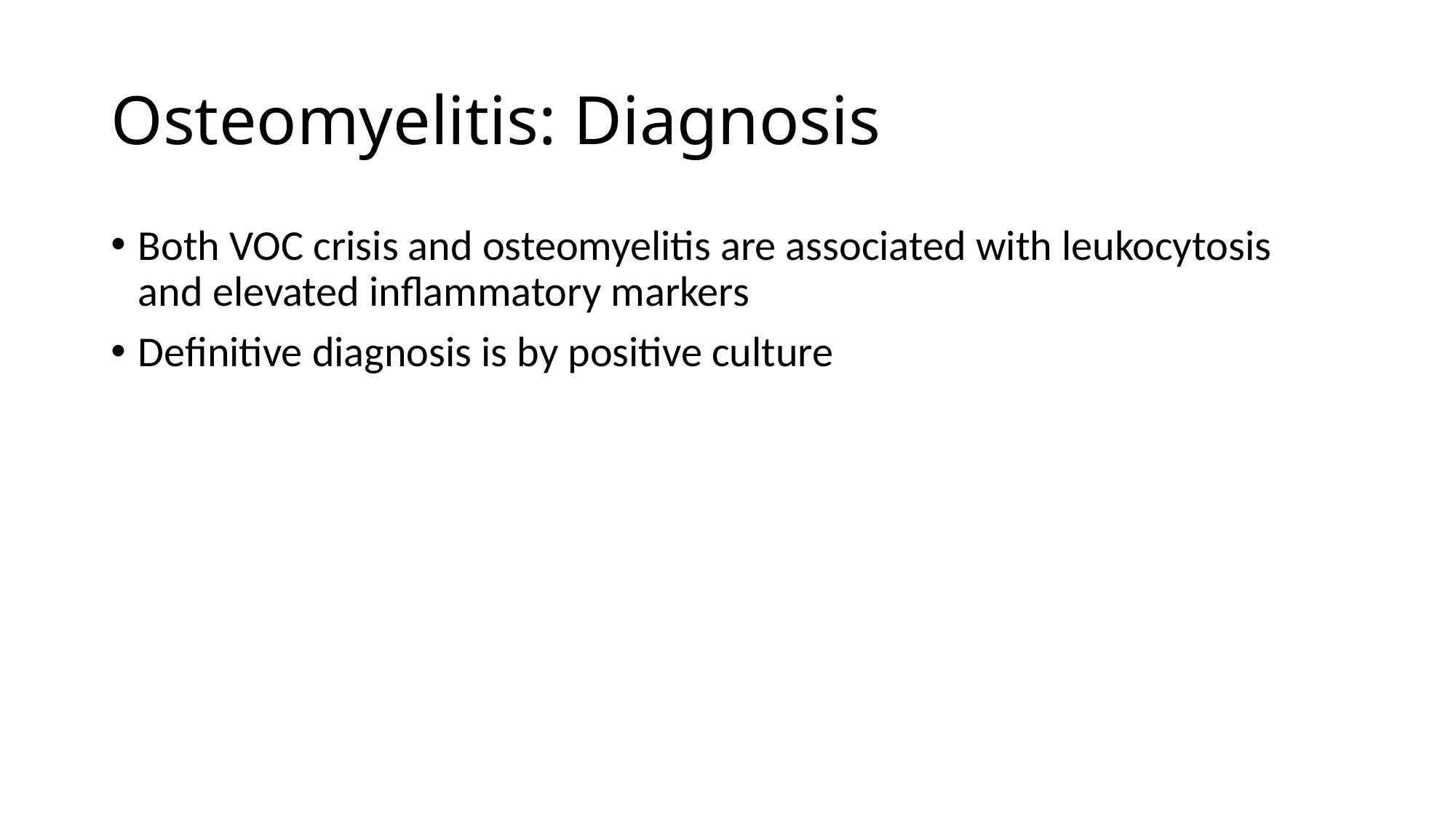

# Osteomyelitis: Diagnosis
Both VOC crisis and osteomyelitis are associated with leukocytosis and elevated inflammatory markers
Definitive diagnosis is by positive culture

## Slide 7
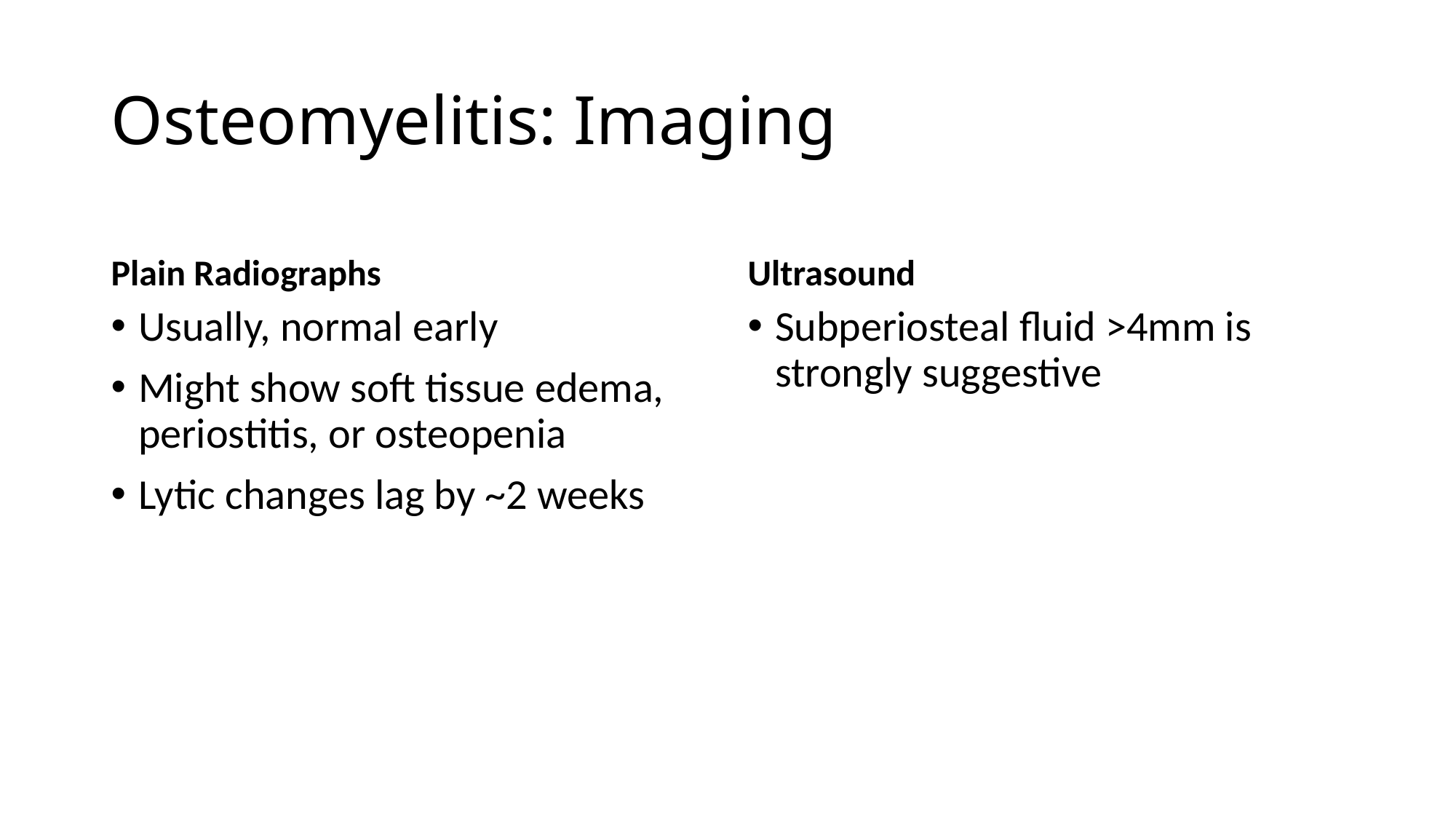

# Osteomyelitis: Imaging
Plain Radiographs
Ultrasound
Usually, normal early
Might show soft tissue edema, periostitis, or osteopenia
Lytic changes lag by ~2 weeks
Subperiosteal fluid >4mm is strongly suggestive

## Slide 8
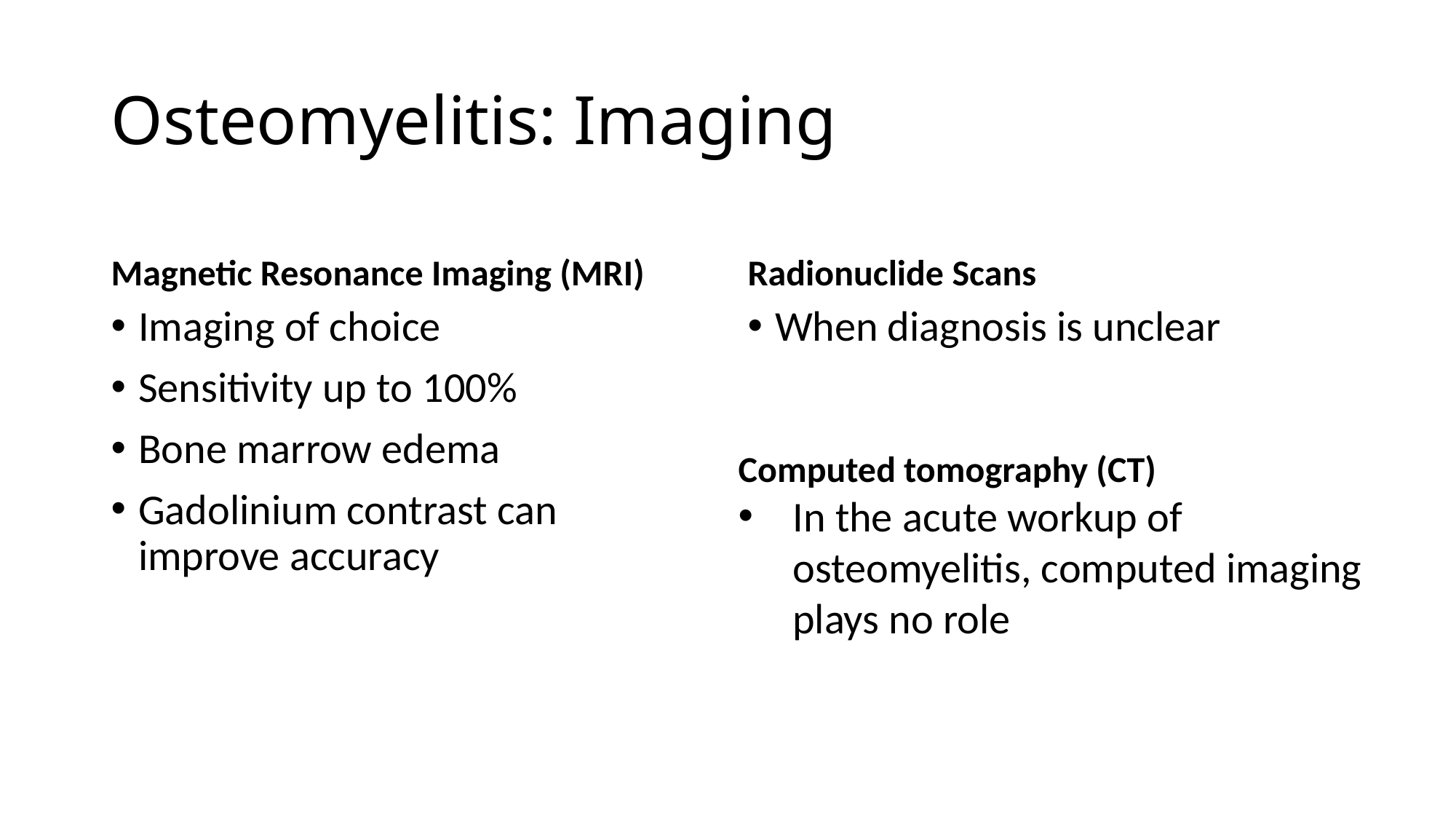

# Osteomyelitis: Imaging
Magnetic Resonance Imaging (MRI)
Radionuclide Scans
Imaging of choice
Sensitivity up to 100%
Bone marrow edema
Gadolinium contrast can improve accuracy
When diagnosis is unclear
Computed tomography (CT)
In the acute workup of osteomyelitis, computed imaging plays no role

## Slide 9
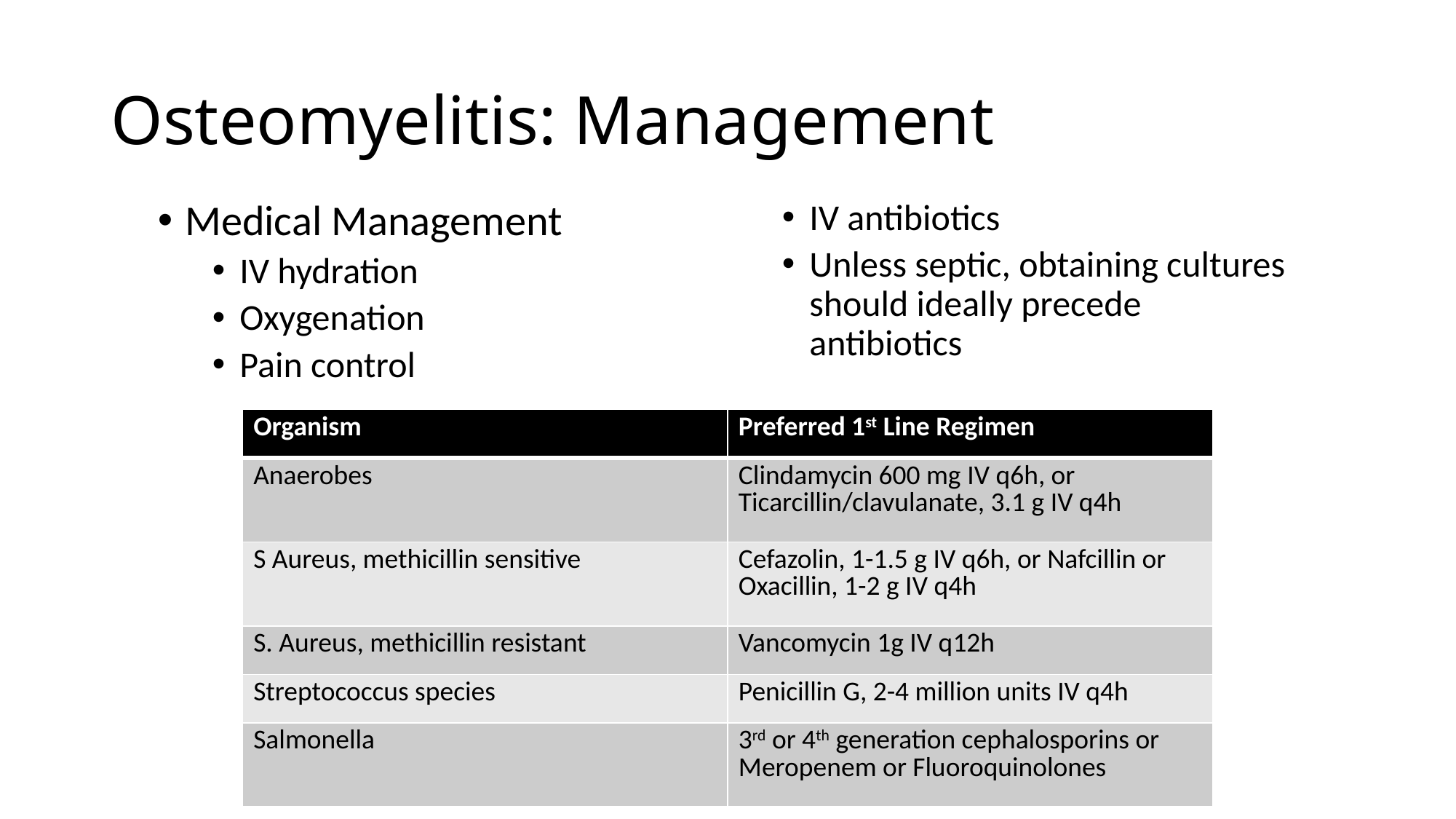

# Osteomyelitis: Management
Medical Management
IV hydration
Oxygenation
Pain control
IV antibiotics
Unless septic, obtaining cultures should ideally precede antibiotics
| Organism | Preferred 1st Line Regimen |
| --- | --- |
| Anaerobes | Clindamycin 600 mg IV q6h, or Ticarcillin/clavulanate, 3.1 g IV q4h |
| S Aureus, methicillin sensitive | Cefazolin, 1-1.5 g IV q6h, or Nafcillin or Oxacillin, 1-2 g IV q4h |
| S. Aureus, methicillin resistant | Vancomycin 1g IV q12h |
| Streptococcus species | Penicillin G, 2-4 million units IV q4h |
| Salmonella | 3rd or 4th generation cephalosporins or Meropenem or Fluoroquinolones |

## Slide 10
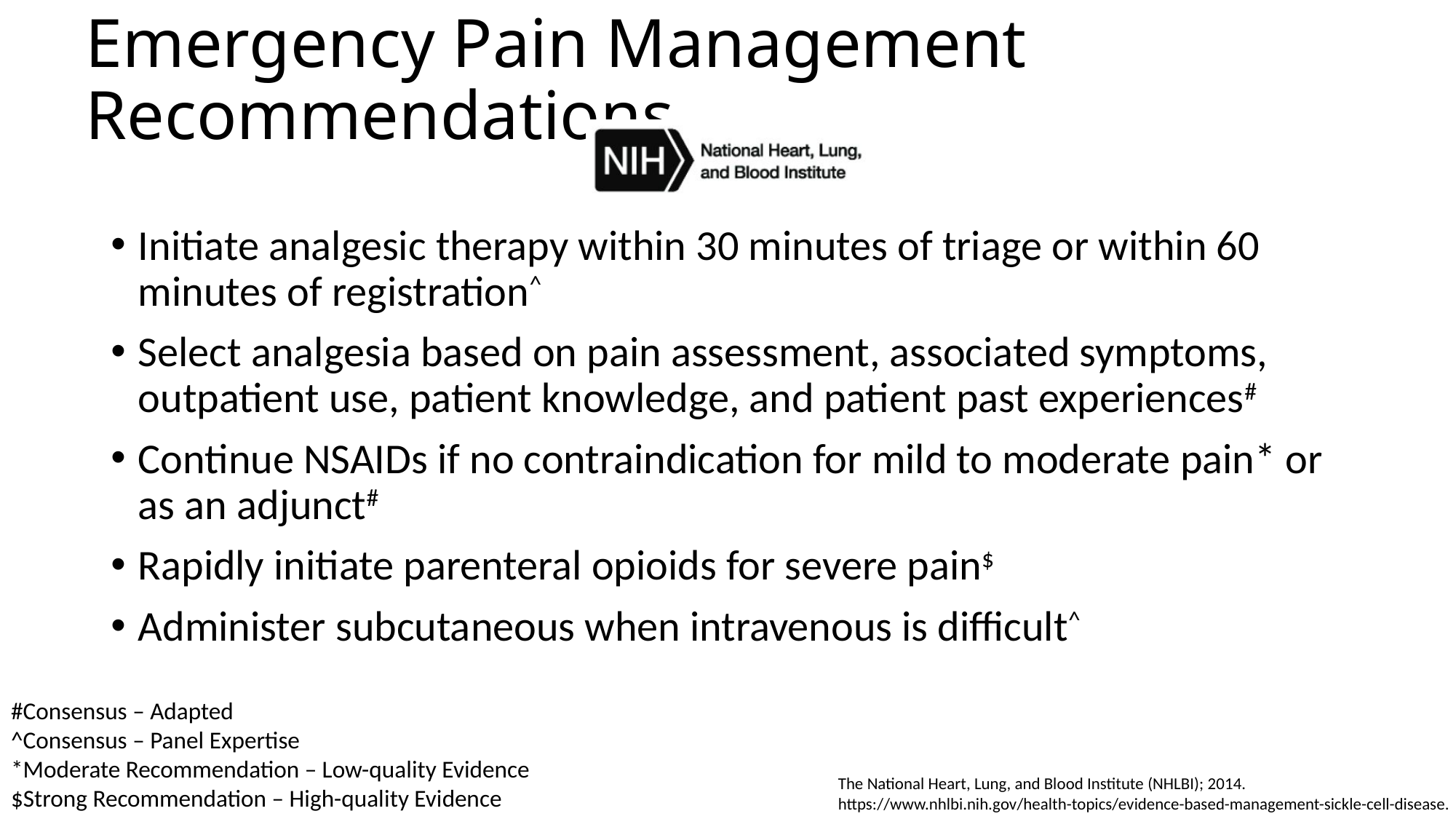

# Emergency Pain Management Recommendations
Initiate analgesic therapy within 30 minutes of triage or within 60 minutes of registration^
Select analgesia based on pain assessment, associated symptoms, outpatient use, patient knowledge, and patient past experiences#
Continue NSAIDs if no contraindication for mild to moderate pain* or as an adjunct#
Rapidly initiate parenteral opioids for severe pain$
Administer subcutaneous when intravenous is difficult^
#Consensus – Adapted
^Consensus – Panel Expertise
*Moderate Recommendation – Low-quality Evidence
$Strong Recommendation – High-quality Evidence
The National Heart, Lung, and Blood Institute (NHLBI); 2014. https://www.nhlbi.nih.gov/health-topics/evidence-based-management-sickle-cell-disease.

## Slide 11
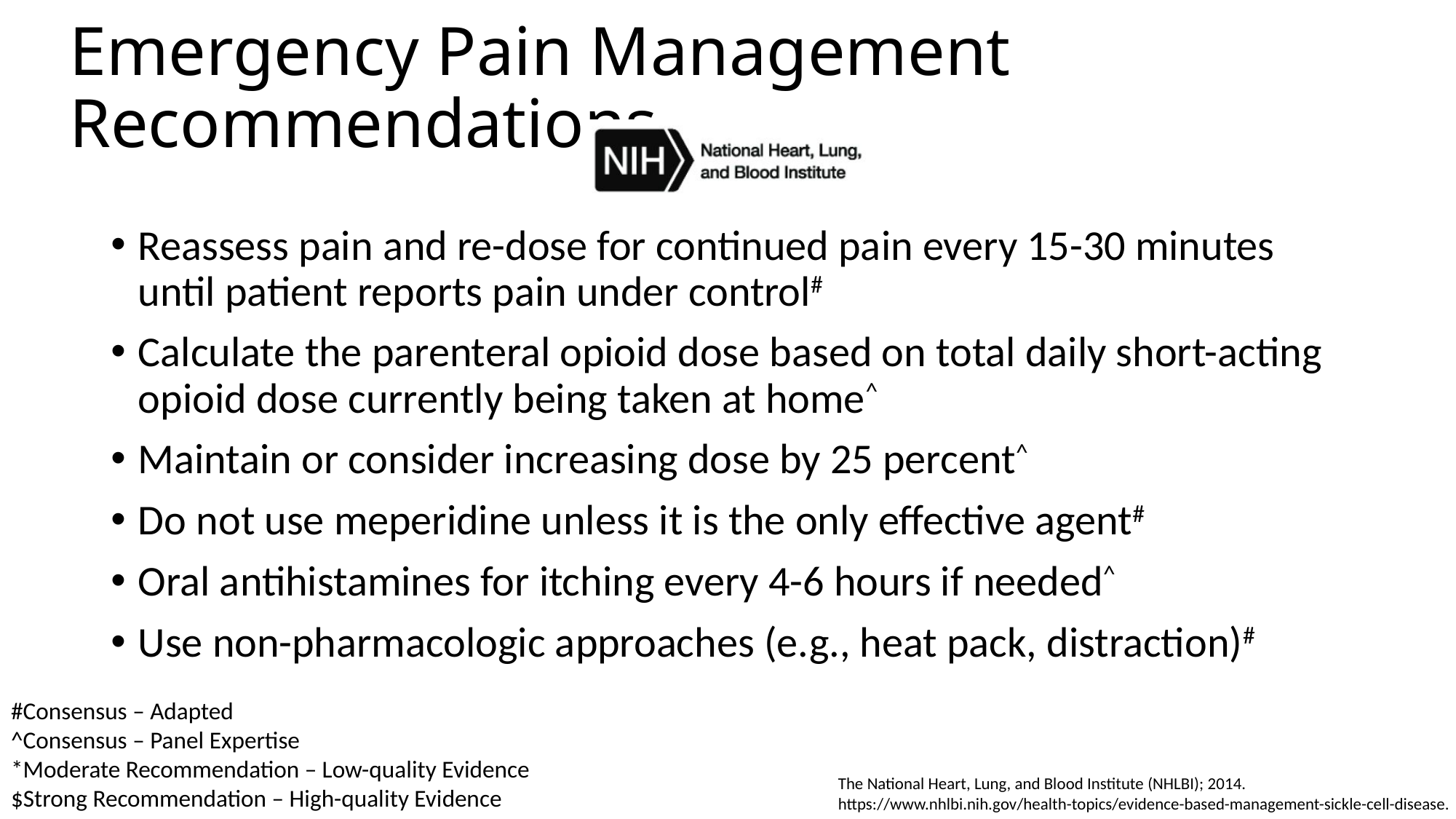

# Emergency Pain Management Recommendations
Reassess pain and re-dose for continued pain every 15-30 minutes until patient reports pain under control#
Calculate the parenteral opioid dose based on total daily short-acting opioid dose currently being taken at home^
Maintain or consider increasing dose by 25 percent^
Do not use meperidine unless it is the only effective agent#
Oral antihistamines for itching every 4-6 hours if needed^
Use non-pharmacologic approaches (e.g., heat pack, distraction)#
#Consensus – Adapted
^Consensus – Panel Expertise
*Moderate Recommendation – Low-quality Evidence
$Strong Recommendation – High-quality Evidence
The National Heart, Lung, and Blood Institute (NHLBI); 2014. https://www.nhlbi.nih.gov/health-topics/evidence-based-management-sickle-cell-disease.

## Slide 12
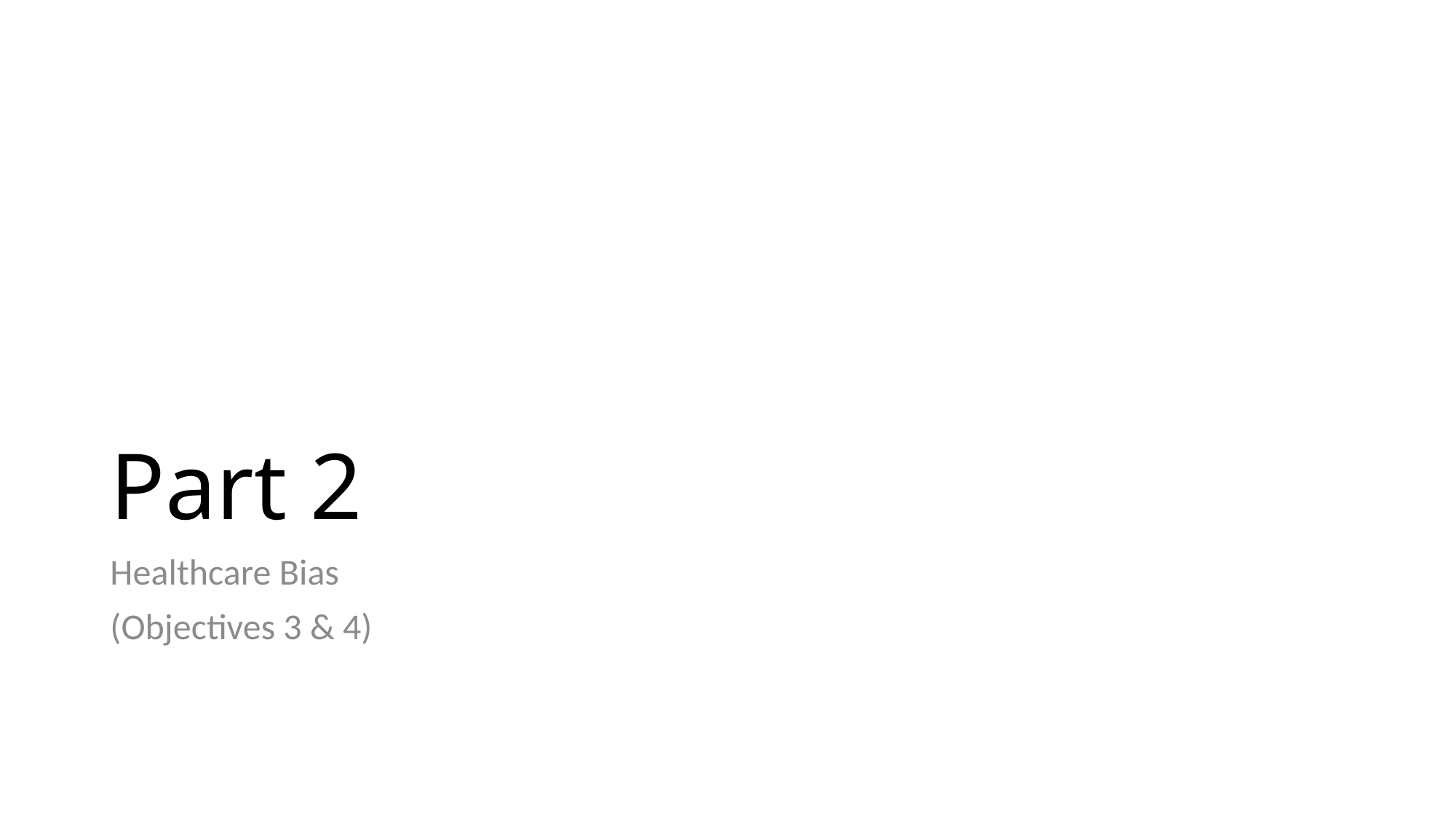

# Part 2
Healthcare Bias
(Objectives 3 & 4)

## Slide 13
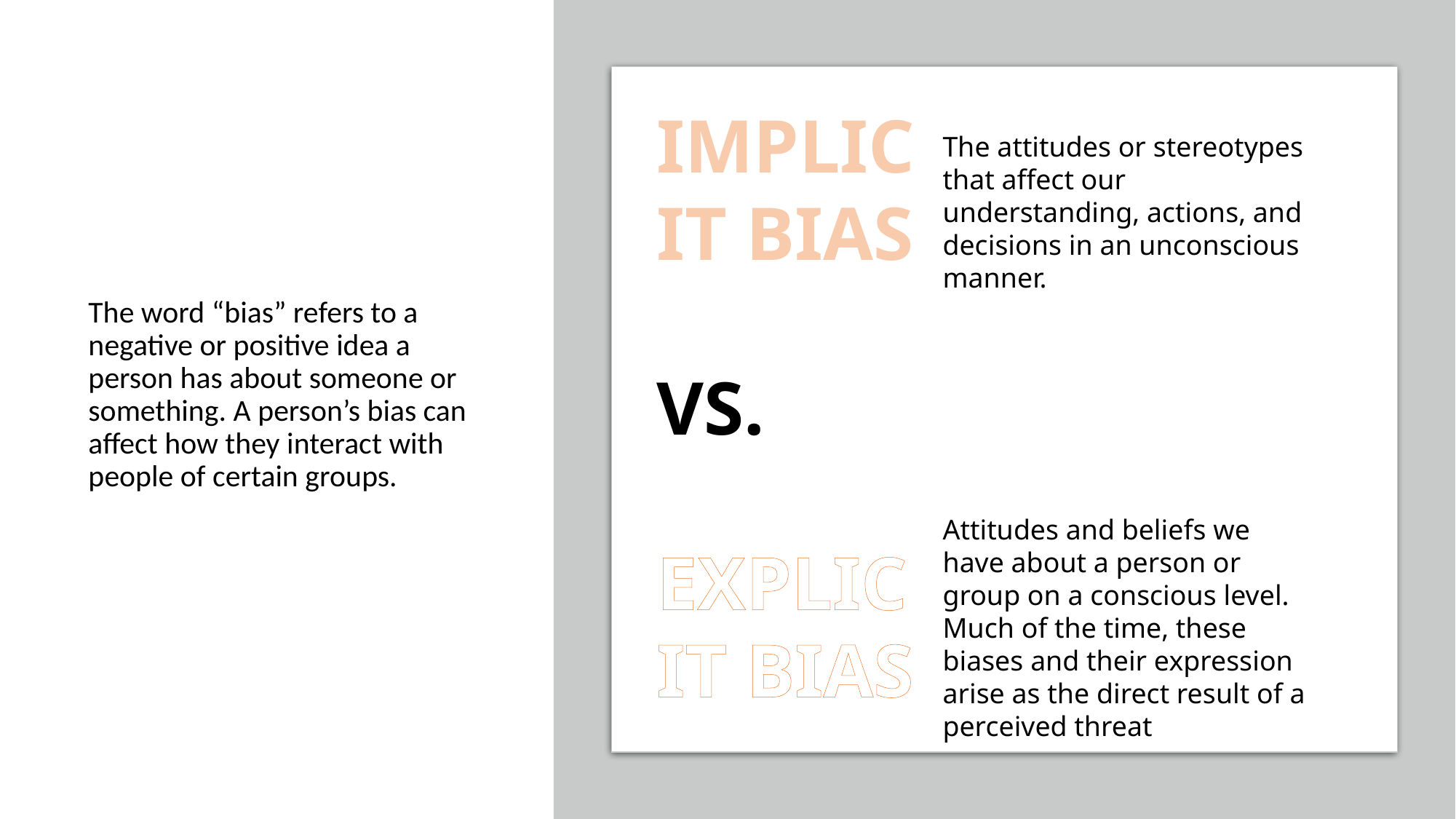

IMPLICIT BIAS
VS.
EXPLICIT BIAS
The attitudes or stereotypes that affect our understanding, actions, and decisions in an unconscious manner.
The word “bias” refers to a negative or positive idea a person has about someone or something. A person’s bias can affect how they interact with people of certain groups.
Attitudes and beliefs we have about a person or group on a conscious level. Much of the time, these biases and their expression arise as the direct result of a perceived threat

## Slide 14
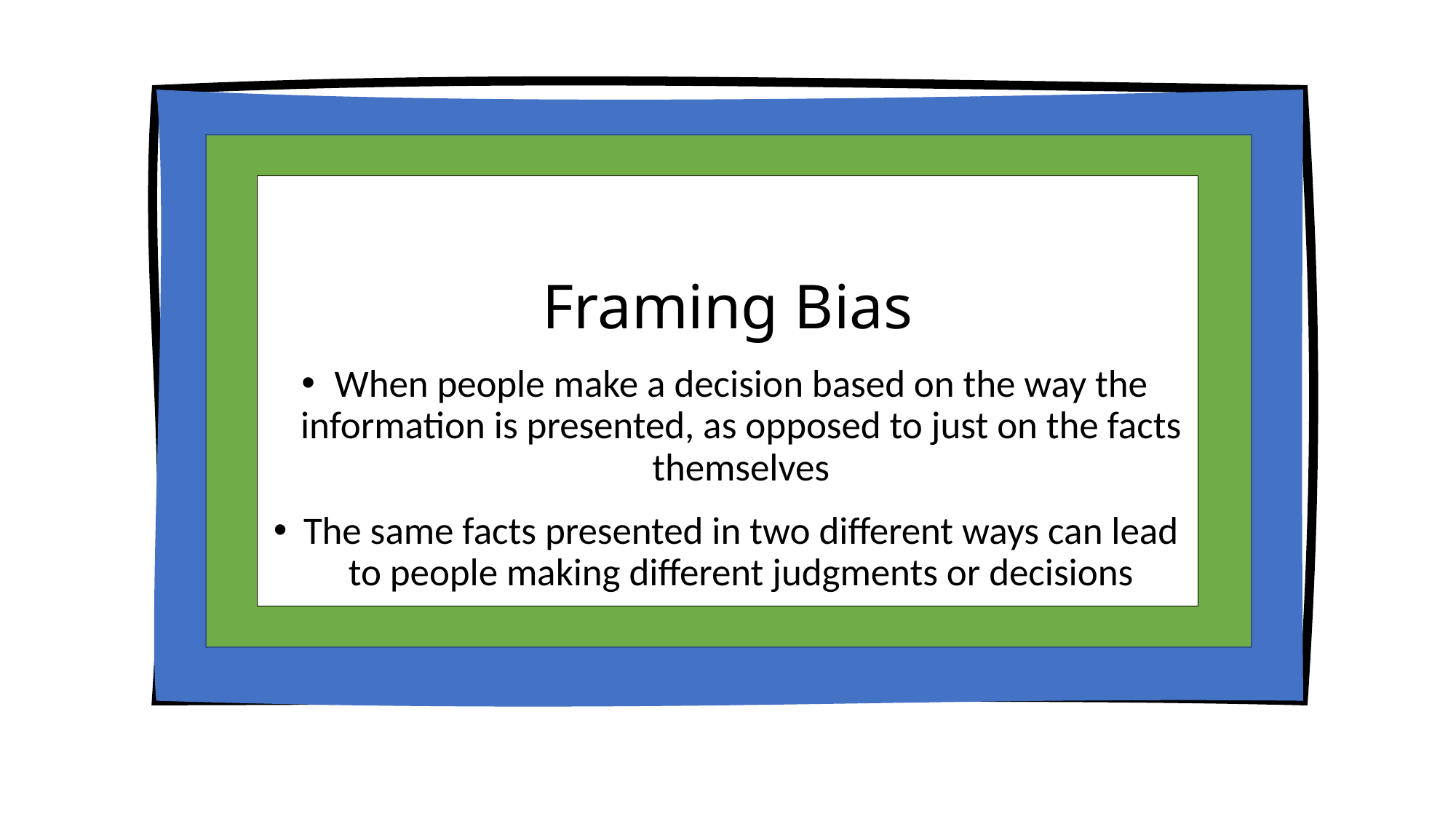

Framing Bias
When people make a decision based on the way the information is presented, as opposed to just on the facts themselves
The same facts presented in two different ways can lead to people making different judgments or decisions

## Slide 15
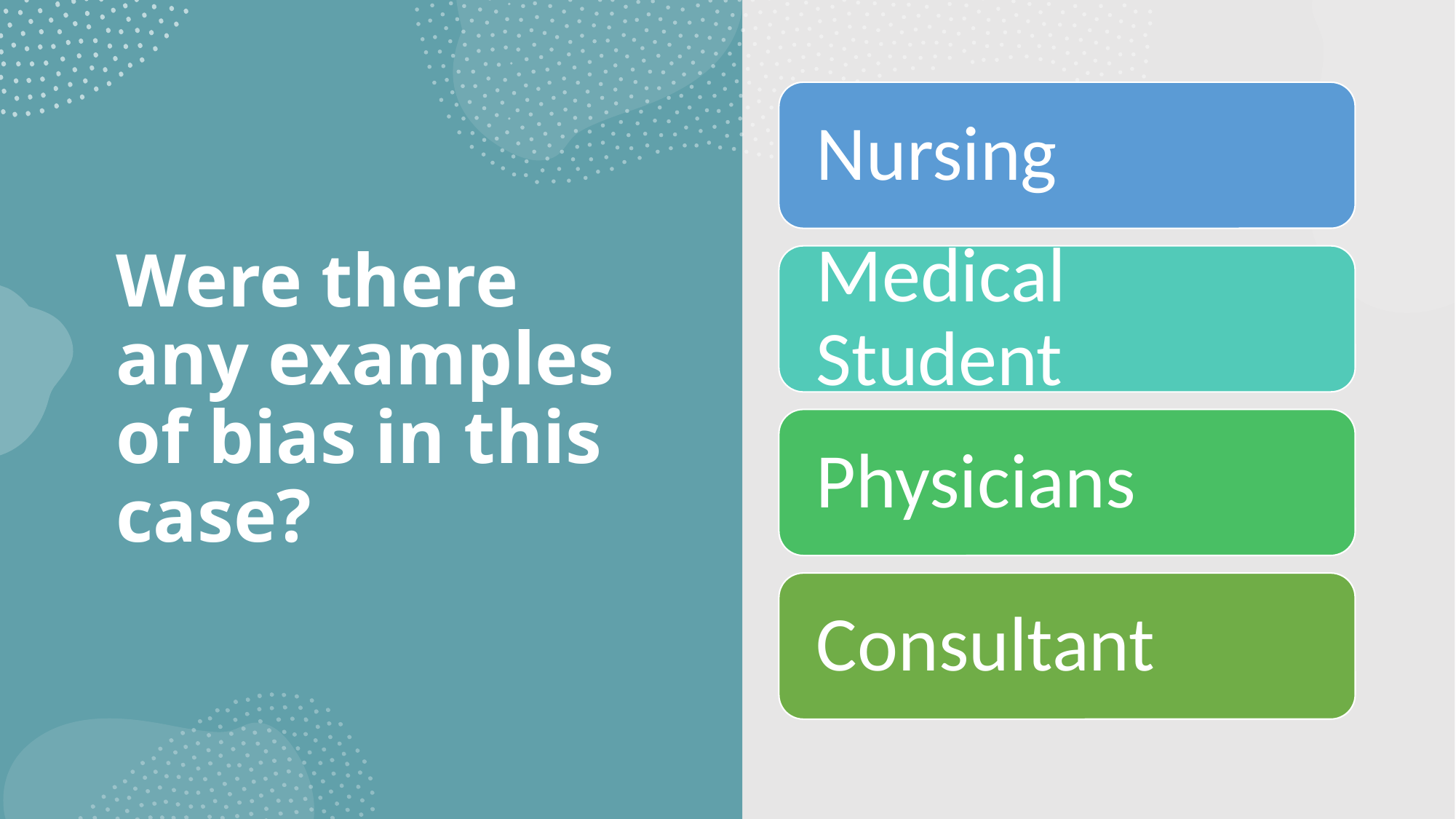

# Were there any examples of bias in this case?

## Slide 16
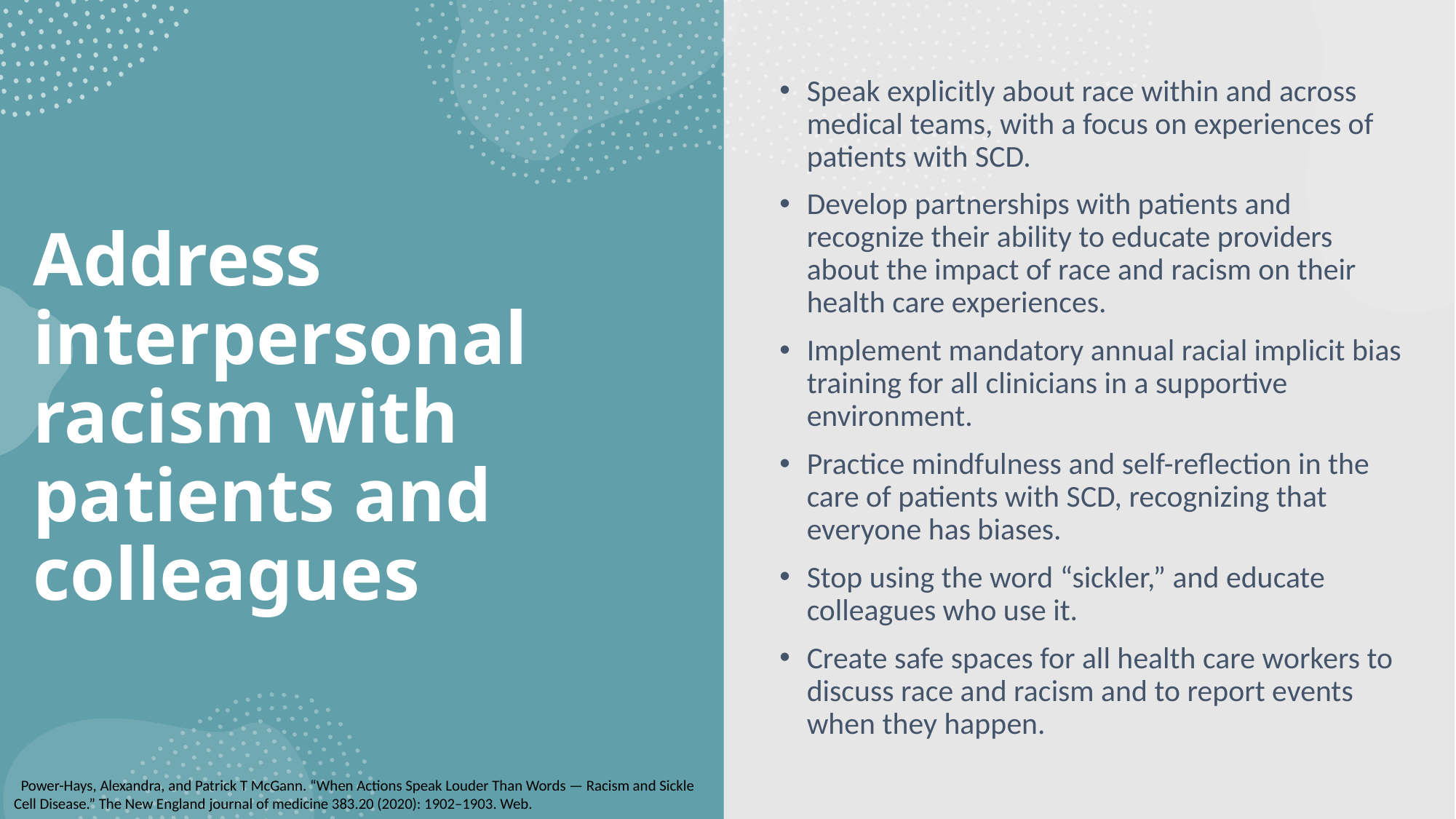

Speak explicitly about race within and across medical teams, with a focus on experiences of patients with SCD.
Develop partnerships with patients and recognize their ability to educate providers about the impact of race and racism on their health care experiences.
Implement mandatory annual racial implicit bias training for all clinicians in a supportive environment.
Practice mindfulness and self-reflection in the care of patients with SCD, recognizing that everyone has biases.
Stop using the word “sickler,” and educate colleagues who use it.
Create safe spaces for all health care workers to discuss race and racism and to report events when they happen.
# Address interpersonal racism with patients and colleagues
 Power-Hays, Alexandra, and Patrick T McGann. “When Actions Speak Louder Than Words — Racism and Sickle Cell Disease.” The New England journal of medicine 383.20 (2020): 1902–1903. Web.
