# Supplementary material for: Not Just a Pain: A Medical Simulation Case About Biased Communication and Osteomyelitis in Pediatric Sickle Cell Anemia
Source: MedEdPORTAL. 2023 Aug 16;19:11335. doi: 10.15766/mep_2374-8265.11335 (PMC10427742; doi:10.15766/mep_2374-8265.11335)
Supplement: Supplementary file 1 — Simulation Case.docxSimulation Stimuli.pptxDebriefing Materials.pptx [file mep_2374-8265.11335-s001.zip › A. Simulation Case.docx]

| **Appendix A: Simulation Case**  SIMULATION CASE TITLE: Not Just a Pain: A Simulation About Biased Communication and Osteomyelitis in Pediatric Sickle Cell Anemia  AUTHORS: Adeola Kosoko, MD, Yakira Alford, MD MPH, Karl Upplegger, MD, Gowri Stevens, MD  **LEARNER AUDIENCE:** emergency medicine residents, pediatric residents, and/or pediatric emergency medicine fellows (PGY 1-6) | |
| --- | --- |
| **PATIENT NAME:** Alicia  PATIENT AGE: 9-years-old  **CHIEF COMPLAINT:** left arm pain  PHYSICAL SETTING: pediatric emergency department – 1) physician workstation and 2) patient bedside | |
|  | |
| **Brief Narrative Description of Case** | Alicia is a 9-year-old girl with history of sickle cell anemia presents to the emergency department with her parent for diffuse pain, but particularly pain in her left upper extremity. Her parent is concerned that the child is in pain. She will be initially evaluated by the medical student who will be generally dismissive of her acute on chronic pain, as will nursing staff. Despite the bias demonstrated against this patient, the resident must discover that the child likely has osteomyelitis of her left elbow and will need optimal medical workup, pain control, and appropriate disposition. The resident should also identify and address bias for optimal management of the patient. |
| **Primary Learning Objectives** | By the end of this activity, learners will be able to:   1. Medically optimize care for a patient with sickle cell anemia and resultant osteomyelitis 2. Appropriately treat pain crisis in a patient with sickle cell anemia 3. Explain the impact of providers’ language biases on patient care 4. Develop strategies for discussing sickle cell anemia as a commonly stigmatized diagnosis in the clinical setting |
| **Critical Actions** | Critical Actions   1. Appropriately treat the patient’s pain, reducing the pain score assessment 2. Identify bias by member(s) of the care team 3. Create a working diagnosis for acute elbow pain 4. Obtain appropriate imaging of elbow 5. Obtain blood cultures 6. Consult orthopedic surgery team 7. Consult hematology team 8. Admit the patient |
| **Learner Preparation or Prework** | None |

| Initial Presentation | | | |
| --- | --- | --- | --- |
| **Initial Vital Signs** | Blood Pressure 128/73mmHg  Pulse 118 beats/min  Respiratory Rate 18 breaths/min  Temperature 38.1°C  Oxygen Saturation 97% | | |
| **Overall Setting and Appearance** | 1) Physician workstation – An area typical of where physicians work away from patients/families. A space where the nurse will notify the physicians about the new patient and the medical student will give a patient presentation based on his/her history and physical exam.  2) Patient bedside - A private emergency room with a child laying on the bed with her parent pacing around the room. The child is laying curled on the bed and watching her tablet computer. | | |
| **Standardized Participants (and Their Roles in the Room at Case Start)** | Nurse: “Sorry I had to make the patient with pain a level 2 ESI, she has sickle cell. She’s in room 2 with her parent.”  Medical Student: “I went to see the patient. Can I tell you about her? I got the history from her parent. The parent didn’t seem too happy that I couldn’t get the patient any medications because I’m a student. But, here goes:  ‘She’s a 9-year-old sickler with HgbSS. She has been having pain the past 3 days and her parent has been giving her ibuprofen and acetaminophen for her pain at home. The pain is mostly in her left arm, but otherwise it’s all over, but mostly in her arms, legs, back, and hips. She has a low-grade fever. I think it’s because she’s wrapped up in her blanket. The child doesn’t really want to talk to me. I don’t think she’s in much pain because she’s just laying there, playing with her tablet. But she did let me examine her. Her exam is pretty unremarkable except for she’s a bit whiny and doesn’t like it when I try to move her left arm. So, my assessment and plan is a 9-year-old sickler with a pain crisis. I think we just need to give her more acetaminophen and ibuprofen and maybe we could check some labs, but mostly, we just need to reassure her mom that she’s okay, but this type of patient is just going to have pain sometimes.’  The group then goes to the patient bedside where the learners may interact with the patient and her parent. The nurse will not be available for bedside nursing. The nurse explains that they have “other priorities” to attend to in the department. | | |
| **HPI** | The patient is a 9-year-old girl with history of SCD presenting with diffuse pain to her arms and legs for the past 3 days. Parent has been giving acetaminophen and ibuprofen at home as prescribed for when she has pain crises. The presentation is similar to other pain crises but the parent thinks that it might be more severe, particularly in her left arm, so they came to the ED. The parent denies any recent illness or fever at home. | | |
| **Past Medical/Surgical History** | **Medications** | **Allergies** | **Family History** |
| hemoglobin SS  last admission for pain 6 weeks ago | hydroxyurea, acetaminophen, ibuprofen, penicillin | none | older brother with hemoglobin SS |
| **Physical Examination** | | | |
| **General** | Alert, withdrawn, laying on the bed playing on a computer tablet | | |
| **HEENT** | Normocephalic, nontraumatic, tympanic membranes normal bilaterally, oropharynx clear, mild icterus bilaterally | | |
| **Neck** | Trachea midline, no lymphadenopathy | | |
| **Lungs** | Clear to auscultation bilaterally | | |
| **Cardiovascular** | Mildly tachycardic, regular rhythm, no murmurs, rubs, or gallops | | |
| **Abdomen** | Soft, nontender, nondistended, no hepatosplenomegaly | | |
| **Neurological** | Tenderness with palpation of left elbow, decreased range of motion of left elbow due to pain | | |
| **Skin** | Cranial nerves 2-12 grossly intact, strength 5/5 at each extremity, sensation grossly intact at each extremity | | |
| **GU** | No rash, mild edema at left elbow | | |
| **Psychiatric** | Deferred | | |

| Instructor Notes - Changes and CASE Branch Points | | |
| --- | --- | --- |
| **Intervention / Time Point** | **Change in Case** | **Additional Information** |
| The learner corrects the nurse or the medical student’s language about the patient. |  | The standardized participant should simply apologize and explain that they didn’t know or didn’t mean harm. |
| The patient is examined. |  | Patient states: “Ouch that really hurts my arm!” |
| The patient is given one dose of analgesia. | The patient and parent will continue to complain about pain until another dose of analgesia is administered. |  |
| Orthopedic Surgery consultation |  | “What does the x-ray show?”  “Did you get an MRI?” |
| Hematology consultation |  | “Has the patient been receiving *all* her medication?”  “We’ll be glad to see her inpatient if you admit her.” |
| If the learners try to discharge the patient, the parent should explain that she is concerned about her daughter. | The child’s pain should be worse. |  |

**Ideal Scenario Flow**

The nurse approaches the resident/fellow to announce that a high-acuity-triaged patient was roomed. The nurse does not feel that the patient or her pain is or should be a priority for care in the emergency department so the nurse will not be available to help with patient care.

The medical student has seen the patient and gives a patient presentation of history, physical, assessment, and plan.

The resident/fellow is expected to be timely in addressing and correcting the language used by the nurse and the medical student about the patient.

The resident/fellow should perform his/her own evaluation of the patient (history and physical) and elucidate that the parent is concerned about the child’s presentation, because the child does not usually complain about pain specifically in one extremity. When examining her left upper extremity, the resident/fellow should be concerned about an abnormal exam.

When developing an assessment and plan, the resident/fellow should recognize that the patient has a fever and mild tachycardia, and a high pain score. The nurse will question if placing an intravenous line (IV) is necessary. The resident/fellow should advocate for placement of an IV for bloodwork and for appropriate pain management. The resident/fellow should be concerned for osteomyelitis. The patient should receive IV analgesics.

The laboratory studies should be concerning for osteomyelitis (elevated erythrocyte sedimentation rate, c-reactive protein, white blood cell count, and elevated platelets). The patient will have a chronic anemia and will have an elevated reticulocyte count. The resident/fellow must obtain a blood culture. The radiograph of the elbow will show mild soft tissue swelling about the elbow, but no joint space widening. The patient should continue to complain of pain and should receive more IV analgesics.

The resident/fellow should consult orthopedic surgery with concern for osteomyelitis and arrange for the patient to receive magnetic resonance imaging (MRI) and/or a biopsy for culture of the affected bone. The resident/fellow should also consult hematology for optimal management of the patient’s sickle cell anemia whilst receiving an inpatient workup for osteomyelitis.

The patient should receive IV antibiotics which would appropriately cover for osteomyelitis in a patient with sickle cell anemia. Her pain should be reassessed. She and her parent should be updated on the findings and plan. She should be admitted to a floor (acute care) bed for continued workup and management.

**Anticipated Management Mistakes**

1. Not recognizing bias in communication or not knowing how to address/correct biased language. Some learners will not recognize the biased language used by nursing and the medical student when describing the patient. This is not unlike the in vivo clinical setting. It is okay if the resident does not identify the biased language, opportunity to debrief on the oversight will be during the debriefing session.
2. Failure to appropriately treat pain. It is not uncommon for medical providers to withhold or not recognize that a patient with chronic pain is requiring analgesia for breakthrough pain. There is also often bias against patients with sickle cell anemia with the assumption that they are abusing the medical system to obtain opiates. It is helpful to use a pain measurement tool to identify whether pain is appropriately being managed. The patient and her parent should regularly explain that she is uncomfortable. The nurse can also help to report abnormal pain scores.
3. Failure to recognize osteomyelitis. Patients with sickle cell anemia are at increased risk for osteomyelitis. Not all pain is due to “simple” pain crises. If osteomyelitis is not considered, the parent should emphasize that this pain is “different.”
4. Incorrect antibiotic coverage. Patients with sickle cell anemia have a higher risk for Salmonella as a cause of osteomyelitis. Clindamycin and Vancomycin are important in covering for methicillin-resistant S. aureus which is a major cause of osteomyelitis in the remainder of the population, but Salmonella coverage can be overlooked by a resident learner. If inadequate coverage, nursing should inquire whether the resident would like to broaden coverage.
